# Aggression on the psychiatric ward: Prevalence and risk factors. A systematic review of the literature

**DOI:** 10.1371/journal.pone.0258346

**Published:** 2021-10-08

**Authors:** Irene Weltens, Maarten Bak, Simone Verhagen, Emma Vandenberk, Patrick Domen, Thérèse van Amelsvoort, Marjan Drukker

**Affiliations:** 1 Department of Psychiatry & Neuropsychology, Maastricht University, Maastricht, The Netherlands; 2 Mondriaan Mental Health Institute, Maastricht / Heerlen, The Netherlands; Radboud University, NETHERLANDS

## Abstract

**Introduction:**

On psychiatric wards, aggressive behaviour displayed by patients is common and problematic. Understanding factors associated with the development of aggression offers possibilities for prevention and targeted interventions. This review discusses factors that contribute to the development of aggression on psychiatric wards.

**Method:**

In Pubmed and Embase, a search was performed aimed at: prevalence data, ward characteristics, patient and staff factors that are associated with aggressive behaviour and from this search 146 studies were included.

**Results:**

The prevalence of aggressive behaviour on psychiatric wards varied (8–76%). Explanatory factors of aggressive behaviour were subdivided into patient, staff and ward factors. Patient risk factors were diagnosis of psychotic disorder or bipolar disorder, substance abuse, a history of aggression, younger age. Staff risk factors included male gender, unqualified or temporary staff, job strain, dissatisfaction with the job or management, burn-out and quality of the interaction between patients and staff. Staff protective factors were a good functioning team, good leadership and being involved in treatment decisions. Significant ward risk factors were a higher bed occupancy, busy places on the ward, walking rounds, an unsafe environment, a restrictive environment, lack of structure in the day, smoking and lack of privacy.

**Conclusion:**

Despite a lack of prospective quantitative data, results did show that aggression arises from a combination of patient factors, staff factors and ward factors. Patient factors were studied most often, however, besides treatment, offering the least possibilities in prevention of aggression development. Future studies should focus more on the earlier stages of aggression such as agitation and on factors that are better suited for preventing aggression such as ward and staff factors. Management and clinicians could adapt staffing and ward in line with these results.

## Introduction

Aggression is a serious problem on psychiatric wards and has large consequences for patients as well as staff working in mental healthcare: aggressive behaviour is an important reason to seclude or restrain a patient [1], but, according to patients, this feels as being controlled and punished with no therapeutic value [2]. Health care workers experience feelings of anger, anxiety and guilt after an aggressive incident [3] and higher levels of burn-out [4].

Development and expression of aggression is generally explained as multifactorial: being environment-related (design of the ward, privacy, locked doors, ambiance, noise level, over-stimulation), mental health care-system related (regional policy, hospital policy, ward rules, attitude towards patients, cultural factors), patient-related (demographics, cognitive and emotional state, malevolence, pathology) and clinician-related (degree of communication, de-escalation skills, attitudes towards aggression, clinicians’ stress level) [5, 6]. Previous reviews on aggression on psychiatric wards were mainly focused on patient-related factors e.g. isolation of high-risk patients who were prone to develop aggression [7–9]. From a prevention perspective, ward and staff factors provide an interesting avenue for prevention of aggression, while treatment of the psychiatric illness is the only patient factor that reduces the risk of aggression development [10]. Nevertheless it remains unclear how the various factors that explain aggression development interact with each other.

The term aggression is ambiguous: multiple interpretations have been found, and it is often used interchangeably with agitation and violence. Agitation is defined in the DSM-5 as “a state of excessive psychomotor activity accompanied by increased tension and irritability” resulting in non-productive and repetitious behaviour” [11]. Generally it is seen as the precursor of aggression [12]. The WHO defines aggression and violence as the same principal: "the intentional use of physical force or power, threatened or actual, against oneself, another person, or against a group or community, that either results in or has a high likelihood of resulting in injury, death, psychological harm, maldevelopment, or deprivation" [13]. The British National Institute for Health and Care Excellence (NICE) guidelines define aggression as: “a range of behaviours or actions that can result in harm, hurt or injury to another person, regardless of whether the violence or aggression is physically or verbally expressed, physical harm is sustained or the intention is clear” [14]. In these definitions agitation, aggression and violence can be understood as a *continuum* of severity, where agitation evolves into aggression and ultimately into violence. Violence differs from aggression by the severity and intentionality of the behaviour.

The vast amount of literature on aggression may reflect that the topic is important and of interest. Reviews on violence on the emergency ward [15], aggression in forensic settings [16] and on interventions to reduce seclusion and restraint [17] have been published earlier. Reviews on the development of aggression in psychiatric hospitals have also been performed before, but they focussed on a single part of aggression development, such as patient factors [7, 8, 18–20] and staff and ward factors that may contribute to aggression development were left out. The review by Dack is an in-depth analysis of patient characteristics, but only included 34 articles, of which 11 were on forensic wards [8]. They included only studies in which an aggressive group of patients was compared to a non-aggressive group and excluded articles in which the number of incidents were reported instead of the number of aggressive patients. The review by Welsh was conducted solely on environmental factors, but also included forensic and prison settings [21].

Of the reviews that addressed all three factors, these either searched studies between 1983 and 2008 and this thus needs updating [16, 22, 23], or they included only a limited number of studies [16, 23], or included also forensic wards [16]. The article by Cutcliffe provides a theoretic basis for integrating and addressing the different factors as used in this current review but is only a descriptive article lacking statistical evidence and a search strategy [24]. Szabo conducted a review in 2015 which was extensive with 120 included studies but had substantial methodological shortcomings such as not providing statistical evidence for the conclusions and no search strategy [25]. The current systematic review is, thus, the first with an extensive search strategy, clear in- and exclusion criteria and, even more important, the first implementing a quality assessment of included studies. Build on the integrational work of Cutcliffe this systematic review seeks to be as complete as possible in addressing the factors at interplay in aggression development on closed psychiatric wards.

This current review is the first with the aim to compile a complete overview of the available knowledge on patient, staff and ward factors that contribute to the development of aggression on a general psychiatric admission ward and underline this with statistic evidence where possible. With those insights more and improved preventative measures can be designed in the future.

## Method

This systematic review was written in accordance with the Preferred Reporting Items for Systematic Reviews and Meta-Analyses (PRISMA) guidelines [26] following an unpublished protocol intended for internal use. The broad definitions and operationalisations of aggression within the spectrum of aggression were all included, such as “violence”, “aggression”, “aggressive behaviour”, “assaults” and “threats”.

### Eligibility criteria

Studies were included if their main objective was to examine the prevalence of aggression or to study factors associated with aggression on adult inpatient wards in a psychiatric hospital. Aggression is distributed on a continuum of severity ranging from agitation to aggression to violence. Therefore, the various stages of the continuum are incorporated. Inclusion criteria were: adult patients (age 18–65 years), inpatient in a psychiatric hospital, measurements of aggression, violence, agitation, assaults or incidents.

Exclusion criteria were: aggression in case of delirium or dementia, forensic wards, the effect of aggression on mental health workers and health care costs, studies on aggression management, studies on rating scales for aggression prediction, papers on new legislation, publication year < 1999, conference abstracts and non-English language.

### Search strategy

Identified studies were conducted between January 1999 and December 2019, using the electronic databases Pubmed and Embase. The following search terms were used: ‘psychiatry’, ‘inpatients’, ‘aggression’, ‘violence’, and ‘agitation’ (see S1 File for the full search strategy). Reviews on this subject were checked for references.

Eligibility assessment was performed independently by 2 reviewers (IW and MB), disagreements were resolved by consensus.

### Quality assessment

Several checklists were available for quality assessment of the articles, the majority being observational research. However, we could not find an instrument that could be used for the articles in the present systematic review. Although Strobe (Reporting of Observational Studies in Epidemiology) and NOS (Newcastle Ottawa Scale) are most-used and recommended, the NOS is outdated and has never been peer reviewed. Strobe is designed to assist authors in writing a paper on a cohort study, but was used as scoring quality because better assessment lists were lacking. As a quality assessment tool it is inefficient and it cannot be used to score case-control studies.

For this reason, 2 existing methodological quality criteria lists (NOS and Strobe) were combined into a new list: the Observational Study Quality Evaluation (OSQE), which also includes all domains of the Risk Of Bias In Non-randomised Studies of Interventions (ROBINS-I), but with less details [27]. Advantages of the OSQE is that the list is short, easy to understand, complete and suitable for cross-sectional studies. OSQE has sufficient reliability (Pearson correlation coefficient 0.82, 0.70 and 0.82, using in duplo rating of cohort (using present OSQE scores), case-control and cross-sectional studies, respectively [27]. The full article can be found in S2 File).

In the present rating, the maximum number of stars is 15 (a higher score represents a better quality), because 13 obligatory and 2 optional items (moderation and sample size) were included and no cut-off score was used. Quality assessment of the selected studies was performed by authors IW and EV and through meetings consensus was reached on the score of the papers. In case of not reaching mutual agreement, the papers were discussed with MB to obtain consensus.

### Data extraction and analyses

Data extracted were: number of included patients, demographic characteristics of included patients or staff (such as gender, age, IQ, level of education, diagnosis, past violence, substance abuse), admission status (voluntary or involuntary or both), method of measuring aggression (by incident forms, scales used, or extracted from patient records) as well as frequency, severity and type of aggression.

The extracted factors contributing to the emergence of aggression were:

- Patient factors: gender, age, diagnosis, severity of psychopathology, history of aggression or violence, (history of) substance abuse, insight, homelessness.
- Ward factors: location, time, bed occupancy, atmosphere of the ward, restrictions and characteristics of the ward.
- Staff factors: mean age of nurses and medical staff, years of experience, gender, quality of the interaction with the patient and the level of staffing.

Extracted data were imported in the database by IW and checked by MB.

Descriptive statistics were applied such as averages and (weigthed) percentages. Due to heterogeneity of the data, a full meta-analysis was not considered feasible.

## Results

### Search outcome

**Fig 1:**
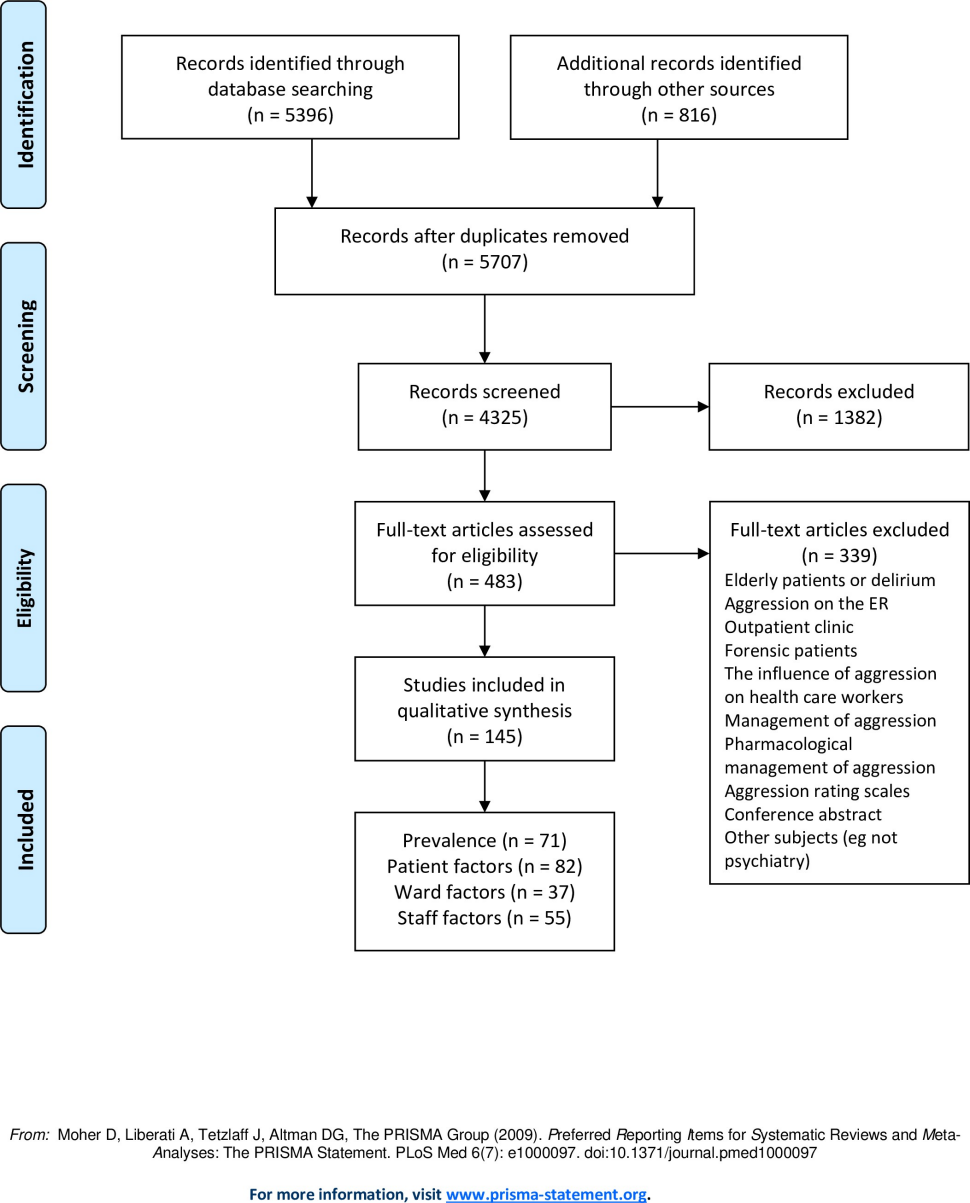
PRISMA flow diagram.

**Fig 2:**
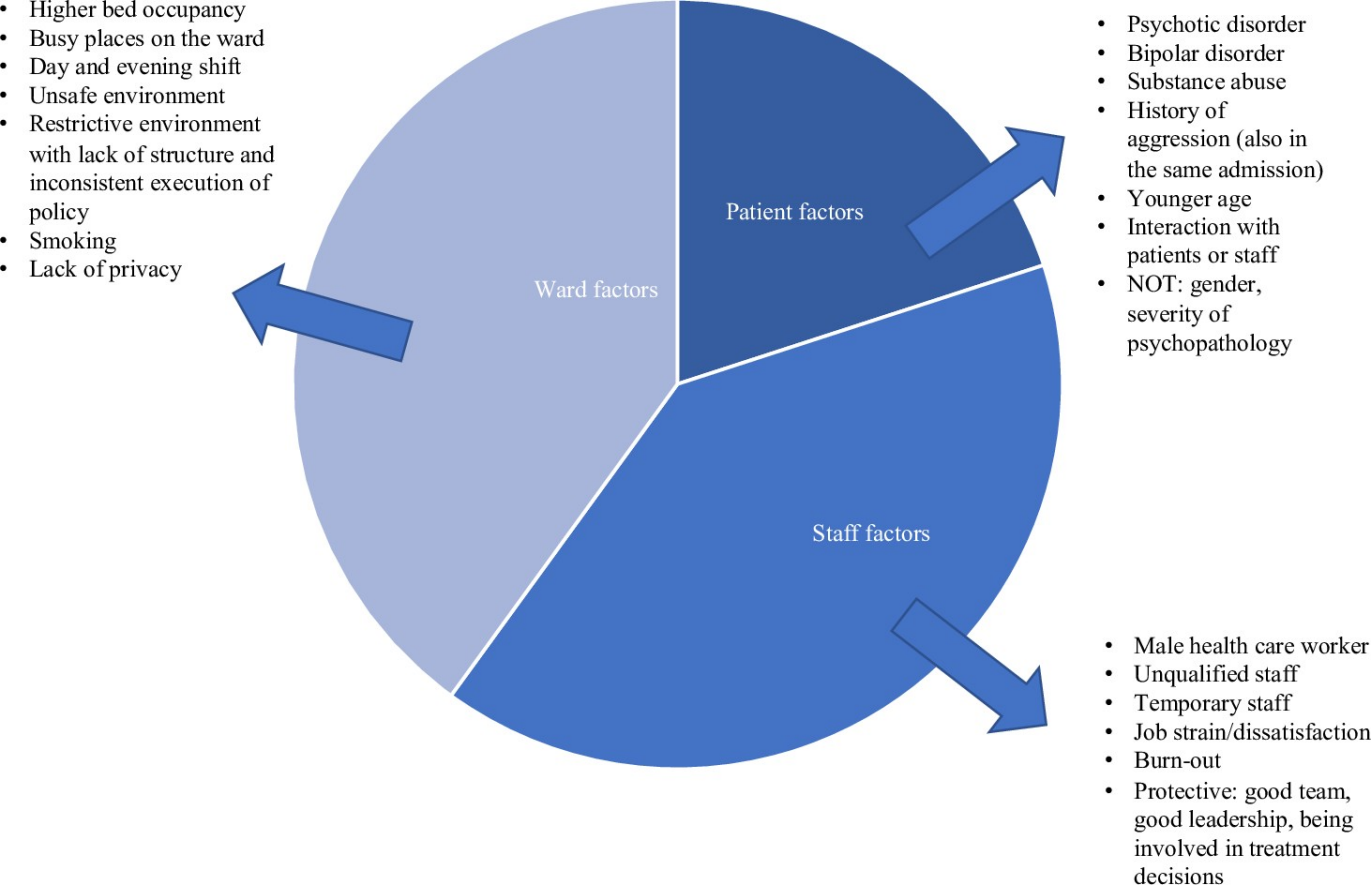
The contribution of the different factors to the development of aggression.

The search yielded 4698 titles from PubMed and 698 titles from Embase. Cross-referencing from 20 reviews published earlier resulted in 816 papers on this topic (Fig 1). Beforehand, studies were divided into four groups (hereafter called factors), representing prevalence, patient factors, ward factors and staff factors. Several articles provided data on 2 or more factors. A large amount of data was subtracted from the studies, which made this review complete, but also complex. Fig 2 depicts a simplified overview of the results that will be presented in this section (Fig 2), in order to prepare the reader for the large body of information that is about to follow. This figure has its limitations, it does not embody the details and considerations described further on, but invites the reader to continue.

### Prevalence of aggression in psychiatric wards

In 71 studies, prevalence rates for inpatient aggression were reported. In this paragraph the results of studies that assessed the prevalence of aggressive incidents by patients on closed wards are presented. A division was made for studies assessing the prevalence of aggression experienced by staff (e.g. percentages of staff experiencing aggression in their career) and aggression incidents in groups of patients (e.g. a percentage of patients admitted that are involved in an incident).

#### Aggression experienced by mental health staff

In 17 studies, prevalence of aggression towards nurses or other members of staff (also named workplace violence) was examined (S1 Table) [28–44]. Prevalence of aggression was measured as the percentage of nurses involved in some sort of aggressive incident. Physical violence, verbal violence, mental abuse, assaults, physical aggression, verbal aggression, threatening (with weapon), physical contact, minor injuries, sexual assault or bullying were included as incidents of aggression.

The studies scored an average of 4.7 (SD = 2.3; range 1–9) on the OSQE. OSQE scores including qualitative comments are available upon request. Of the included staff members, 62% was female. Prevalence of any sort of aggression ranged from 65% [45] tot 99% [42]. When only physical aggression was reported the prevalence ranged from 38% [45] tot 82% [29]. The weighted mean percentage of the prevalence of verbal and physical aggression is 54% (SD = 0.008).

#### Aggression measured in patients

There were 53 patient cohort studies that reported on aggression prevalence in individual patients (S2 Table) [46–95]. Patients were admitted for a duration of 11.6 days to 3 years on a psychiatric ward. The percentage of involuntarily admitted patients ranged from 21.5% to 100%. The type of aggression studied varied from only verbal aggression, to both verbal and physical aggression and some studies also included self-harm.

Percentages of in-patients that were involved in aggression ranged from 7.5% up to 75.9%. The weighted mean percentage of patients that were involved in aggression is 23% (SD = 0.003).

In the first 24 hours or first week, the highest percentages of aggression were measured [53, 72, 79, 81]. Only a minor proportion of the admitted patients were involved in the majority of incidents [51, 54, 56, 62, 72, 78, 85–88, 90, 93].

### Patient factors associated to aggression

Of the 145 included studies, 82 presented data on patient factors associated with aggression. The factors addressed were diagnosis or severity of psychopathology, gender, age, history of violence, substance abuse, homelessness and illness insight. An additional 44 studies reported on other patient factors, which are mentioned at the end of this paragraph.

#### Diagnosis/severity of psychopathology

Of the 68 studies on this item, the average OSQE score was 7 (SD = 2.2; range 2–12) [50, 52, 53, 57, 60, 61, 65, 66, 69, 70, 72, 73, 75, 77, 78, 81, 86–88, 90–92, 96–104], which are presented in Table 1. Psychotic disorder was overrepresented with a range of 29–100%. Conclusion from these data is that patients with a diagnosis of a psychotic disorder (or schizophrenia), a manic episode or bipolar disorder, mental retardation and personality disorder show an elevated risk of becoming aggressive on the psychiatric ward.

**Table 1:** Included studies on the possible association of patient diagnosis and the development of aggression.

Author	Diagnosis	Risk for aggression
Schaefer et al., 2016 [97]	Psychotic disorder	OR = 2.08 (95% CI = 1.58–2.72) p < .05
	Manic disorder	OR = 4.22 (95% CI = 2.99–5.96) p < .05
Johnson et al., 2016 [98]	Bipolar disorder in combination with positive drug screen for cannabis	More episodes of aggression (p = .033)
Van Dongen, 2016 [96]	Persecutory ideation	Predictor for observed aggression on SDAS: B = 0.02 (β = 0.46, t(32) = 2.67, 0.12)
Cho et al., 2014 [52]	Bipolar diagnosis	39.7% of aggressive patients had this diagnosis vs 27.7% in the non-aggressive group (χ^2^ = 8.9; df = 3 p < .05)
Van Dongen, 2012 [57]	Persecutory ideation	Risk for aggression: r = 0.47 (p *=* .01)
	Delusional distress	Risk for aggression: r = 0.44 (p = .003)
Kruger & Rosema, 2010 [61]	Mental retardation	More often diagnosed in violent patients (p = .0017)
	Schizophrenia	Smallest proportion of this diagnosis in violent patients (no statistics provided)
	Disorganised behaviour	Seen less in violent patients (p = .0214)
Biancosino et al., 2009 [65]	Schizophrenia	OR = 3.25 (95% CI = 1.11–9.57) p = .032
	Bipolar disorder	OR = 4.61 (95% CI = 1.51–14.06) p = .007
	Personality disorder	OR = 5.89 (95% CI = 1.9–18.23) p = .002
	Mental retardation	OR = 6.78 (95% CI = 1.32–34.91) p = .022
Bilgin, 2009 [100]		Percentage of physically aggressive patients per diagnostic group:
	Schizophrenia	34%
	Alcohol-substance use disorder	22%
	Bipolar disorder	24%
Carr et al., 2008 [66]	Schizophrenia	OR = 1.8
	Depression	OR = 0.57
	Personality disorder	AOR = 2.67
Abderhalden et al., 2007 [72]	Schizophrenia	OR = 2.10 (95% CI = 1.54–2.88) p < .001
	Substance use disorder	OR = 0.50 (95% CI = 0.33–0.76) p < .001
	Gender and affective disorder	OR = 0.68 (95% CI = 0.43–1.08) p = .101
	Neurotic and personality disorder	OR = 0.56 (95% CI = -.35–0.90) p *=* .002
Ketelsen et al., 2007 [69]	Schizophrenia	OR = 2.85 (95% CI = 1.94–4.20) p < .001
El-Badri & Melsop, 2006 [73]		Percentage of aggressive patients per diagnostic group:
	Schizophrenia	48%
	Bipolar disorder	27%
	Substance misuse	14%
Raja & Azzoni, 2005 [102]	In violent group diagnoses that were more likely:	
	Schizophrenia	χ^2^ = 14.9; df = 2; p *<* .001
	Mania	χ^2^ = 8.4; df = 2; p *<* .001
Mellesdal, 2003 [81]	Schizophrenic disorder	OR = 1.93 (95% CI = 1.29–2.90)
Chou et al., 2002 [104]	Psychotic disorder	OR = 2.07 (95% CI = 1.34–3.22)
Omerov et al., 2002 [103]		Percentage of violent patients per diagnostic group:
	Schizophrenia	36%
	Other psychotic disorder	18%
	Bipolar disorder	17%
Grassi et al., 2001 [87]	Schizophrenia or delusional disorder	More frequent diagnosis in violent patients: χ^2^ = 17.5; df = 2; p < .001
Soliman & Reza, 2001 [86]	Diagnoses that were more likely to be in the violent group were:	
	dissocial and emotionally unstable personality disorders	χ^2^ = 16.10; p < .001
	personality disorder with non-schizophrenia diagnosis	χ^2^ = 16.22; p < .001
	non-alcohol substance use or dependence with nonschizophrenia diagnosis	χ^2^ = 18.73; p < .001
Barlow et al., 2000 [91]		Mental state was a causal factor in 65.71% of the cases
		In group of 60 aggressive patients 53.3% had a presence of hallucinations or delusions
	Schizophrenia	OR = 1.96 (95% CI = 1.38–2.79)
	Bipolar disorder	OR = 2.81 (95% CI = 1.72–4.56)
	Depression	OR = 0.44 (95% CI = 0.26–0.74)
	Adjustment disorder	OR = 0.54 (95% CI = 0.33–0.87)

First colum is first author and year of publication and reference, SDAS: Social Dysfunction and Aggression Scale, OR: Odds Ratio, AOR: Adjusted Odds Ratio, CI: 95% Confidence Interval, B: regression coefficient, b: standardised regression coefficient, t: t-score, c^2^: Chi-square, df: degrees of freedom, r: correlation coefficient.

In one study aggressive patients were distracted by internal stimuli during an incident [105] in 60% of the incidents. This statement was studied in more detail in an ecological momentary assessment (EMA) study among 27 patients with schizophrenia, bipolar disorder or schizoaffective disorder: a positive association was found between physical aggression and symptomatology as recorded by patients on the EMA: suicidal ideation (Odds Ratio (OR) = 11.59; 95% CI = 2.95–45.58), being spied upon (OR = 4.27; 95% CI = 1.34–13.61), thought insertion (OR = 5.06; 95% CI = 1.51–16.96), delusional thoughts (OR = 12.39; 95% CI = 2.47–62.04), feeling angry (OR = 3.64; 95% CI = 1.23–10.77), feeling sad (OR = 11.96; 95% CI = 2.67–53.51), feeling restless (OR = 3.41; 95% CI = 1.04–11.17) [49].

In 12 papers diagnosis had no significant contribution to the development of aggression [50, 53, 60, 70, 75, 77, 78, 88, 90, 92, 99, 101].

Assessment of symptom severity in relation to aggression development was addressed in 14 studies comparing PANSS or BPRS scores (as a measure for symptom severity) between aggressive and non-aggressive patient groups (see Table 2) [47, 48, 67, 70, 76, 79, 88, 94, 102, 106–110]. For the PANSS and the BPRS mixed results were found between the score on the questionnaire and the development of aggression: both a positive correlation (a higher score implied a higher risk of aggression) as well as no correlation of the severity of psychopathology on the development of aggression was found, which was also seen in different subscales. The subscale depression/anxiety showed higher symptom scores in non-aggressive patients, with OR = 0.78; 95% CI = 0.65–0.95.

**Table 2:** Included studies with an association between PANSS(-EC)/ BPRS scores and the development of aggression.

Author	Study group	PANSS(-EC)/BPRS
Sacchetti et al., 2018 [106]	Severely agitated patients	Higher scores on PANSS: 82.72 vs 87.54 (p < .01)
Menculini et al., 2018 [47]	Aggressive patients	Higher PANSS-EC scores: 13.56 (4.81) vs 10.94 (3.95); p *=* .012
		PANSS-EC: OR = 1.14 (95% CI = 1.01–1.28)
		Lower PANSS-anxiety/depression scores: 6.08 (2.20) vs 7.53 (3.21); p *=* .027
		PANSS anxiety/depression: OR = 0.78 (95% CI = 0.64–0.95)
Mi et al., 2017 [48]	Agitated vs non-agitated group	PANSS-EC: 20.39 vs 11.47; χ^2^ = 29.66
Zhu & Wang, 2016 [107]	Aggressive patients vs non-aggressive patients	Regression analysis: antisocial factor: OR = 2.1 (95% CI = 1.26–3.50), impulsiveness OR = 7.134 (95% CI = 2.96–17.21) and depression OR = 1.291 (95% CI = 1.07–1.56)
Amore et al., 2008 [67]	Patients with physical aggression before admission	Higher BPRS total scores: 51.8 vs 44.8; p < .05 and higher scores on thought disturbance factor of the BPRS: 13.9 vs 10.7; p < .05
		Thought disturbance factor on BPRS: OR = 1.097 (95% CI = 1.005–1.197) p < .05
		Hostility suspiciousness factor on BPRS: OR = 1.14 (95% CI = 1.08–1.21) p < .001
		Activation on BPRS: OR = 1.14 (95% CI = 1.05–1.22) p < .01
Cohen et al., 2008 [108]	Aggressive vs non-aggressive patients	BPRS total: no significant difference
		Non-aggressive group: higher scores on depression/anxiety scale *p* < .05 and lower score on hostile/suspiciousness scale: p < .05
Goldberg et al., 2007 [70]	Aggressive vs non-aggressive patients	PANSS total score: not significantly different between groups
		Positive symptom subscale of PANSS: 19 vs 15.27 F 3.6; p *=* .03
Verma et al., 2005 [109]	Aggressive vs non-aggressive patients	Hostility on PANSS: 4.1 vs 2.2; p *=* .02
		Poor impulse control on PANSS: 3.8 vs 1.6; p *=* .001
Raja & Azzoni, 2005 [102]	Non-aggressive patients, hostile patients and violent patients	BPRS total: no significant difference
		BPRS hostility: F = 138.93; df 1278; p *<* .001
		In hostile group: higher BPRS psychotic cluster score: F = 11.85; df1278; p *<* .001
		Non-hostile group:
		More depression: χ^2^ = 27.3; df = 2; p *<* .001
Nolan et al., 2005 [76]	Aggressive patients	PANSS subscales significantly higher: hostility (difference of 0.69 points; p = .026) and poor impulse control (difference of 0.92 points; p *<* .0001).
		Total PANSS score within 3 days of the incident is higher than outside this window of 3 days: F = 26.95; df1.981; p *<* .001
Krakowski & Czobor, 2004 [110]	Violent vs non-violent patients	The frequency of physical assault was correlated with total BPRS score: F = 5.39; df = 1; p = .02
Troisi et al., 2003 [79]	Aggressive patients	Total BPRS: not significant
		Hostility on BPRS: not significant
		Tension-excitement on BPRS: OR = 1.96 (95% CI = 1.17–2.45) p = .005
Ehmann et al., 2001 [88]	Violent vs non-violent patients	PANSS total: OR = 1.03; p = .13
Arango et al., 1999 [94]	Aggressive patients	Total PANSS score: t = -3.97; df = 58; p *<* .001
		Subscales PANSS:
		• suspiciousness: z = -2.34; p *<* .05
		• hostility: z = -3.55; p *<* .001
		• hallucinatory behaviour: z = -1.8; p = .07
		• uncooperativeness: z = -4.16; p *<* .001
		• lack of insight: z = -2.72; p *<* .01
		• poor impulse control: z = -4.12; p *<* .001

First colum is first author and year of publication and reference, PANSS: Positive and Negative Symptom Scale, PANSS-EC: Positive and Negative Symptom Scale-Excited Component, BPRS: Brief Psychiatric Rating Scale, OR: Odds Ratio, CI: 95% Confidence Interval, t-score, c^2^: Chi-square, df: degrees of freedom, F: ANOVA test statistic, z: standard score.

Higher scores on PANSS or BPRS appear to increase the risk for aggression, whereas a high score on the subscale for depression may be protective for aggression.

#### Gender

Thirty-seven studies reported on gender as a potential factor related to aggression development [52–54, 59, 61, 62, 65–67, 69, 70, 72–74, 77, 78, 81, 83, 88, 90–92, 94, 99–102, 108–117]. These studies had an average OSQE score of 7.4 (SD = 1.9; range 4–12) with a total of 25,611 patients included, predominantly males (59%; SD = 16.6; range 0%-86%). Results were inconclusive: in 22 studies gender was not significantly associated with aggression [53, 59, 61, 62, 67, 70, 74, 77, 78, 81, 90–92, 94, 99, 101, 102, 108, 109, 112–114], on the other hand in three studies females showed more aggression [83, 88, 110], whereas in the remaining 12 studies males showed more aggression [52, 54, 65, 66, 69, 72, 73, 100, 111, 115, 116, 117] (see Table 3).

**Table 3:** Included studies on the possible association of gender and aggression development in patients.

Author	Gender as factor in the development of aggression
Meng et al., 2018 [111]	72.4% male vs 27.6% female (p *<* .05) in aggressive group
Sehlo et al., 2015 [115]	Male sex: OR = 1.27, (95% CI = 1.10–1.84) p = .04
Cho et al., 2014 [52]	33.6% males and 23.9% females with aggression
Stewart & Bowers, 2013 [54]	More incidents of anger in males 0.19 (*SD* = 0.54) vs females 0.09 (*SD =* 0.34) t = 2.46 (p = .014)
	Male patients: more incidents of threats: 0.57 (*SD* = 2.69) vs 0.34 (*SD =* 2.64) in females (*p* = .053)
Bilgin, 2009 [100]	Verbal aggression experienced by nurse: 46% by male patient.
	Physical aggression experience by nurse: 24% by male patient.
Biancosino et al., 2009 [65]	More males in hostile and violent group: χ^2^: 8.02 (p = .018)
Carr et al., 2008 [66]	Same amount of admission of males and females had incident of aggression.
	Threatening behaviour was found more in males than females: 20% vs 10.9% (χ^2^ = 23.48; p *<* .001)
Ketelsen et al., 2007 [69]	Male gender: 61.4% of aggressive patients (χ^2^ = 2.6; p = .11)
Abderhalden et al., 2007 [72]	Male gender: OR = 1.21 (95% CI = 0.92–1.60) p = .18
El-Badri & Mellsop, 2006 [73]	2% of male and 7% of female patients were aggressive
	χ^2^ = 26.67; df = 1; p *<* .001
Waldheter et al., 2005 [116]	Males exhibit more severe violence: β = -0.460 (p *<* .01)
Krakowski & Czobor, 2004 [110]	More physical assaults in women (F = 3.45; df = 1; p = .06)
Bowers & Alexander, 2003 [83]	Female gender had higher frequency of events
Ehmann et al., 2001 [88]	Female gender OR = 17.12; p = .02
Steinert et al., 1999 [117]	Of the 65% of patients that were aggressive 75% was male and 53% was female. χ^2^ = 7.845; df = 1; p = .005

First colum is first author and year of publication and reference, OR: Odds Ratio, CI:95% Confidence Interval, β: standardized regression coefficient, χ^2^: Chi-square, df: degrees of freedom, SD: Standard Deviation, F: ANOVA test statistic.

#### Age

In 35 included studies, age was examined in association with aggression with an average OSQE score of 7.7 (*SD* = 1.8; range 4–12) [52–54, 59, 61, 62, 65–67, 69, 70, 72–74, 78, 79, 81, 83, 87, 88, 90–92, 94, 97, 99, 101, 102, 106, 109, 110, 112–114, 118]. The number of patients included in these studies was 30,143.

Age was not associated with the development of aggressive behaviour in 23 studies [53, 61, 62, 67, 70, 73, 74, 79, 81, 88, 90–92, 94, 97, 99, 101, 109, 112–114]. In the other 12 studies, younger age was predominantly found to be a factor contributing to the appearance of aggression. See Table 4 for the results for age. The exact definition of younger age is not clearly stated in all articles; where it is described it is shown in the Table. The interpretation of age is based on crude information on the mean ages given in the literature. Age does not seem to be a clear factor to contribute to aggression development.

**Table 4:** Included studies on a possible association between age and aggression development in patients.

Author	Age influencing the development of aggression
Suchting et al., 2018 [118]	Younger age is predictive of aggression
Sacchetti et al., 2018 [106]	Severely agitated patients are younger: 42.14 years (*SD* = 13.52) vs 38.27 years (*SD* = 11.39) p<0.001 compared to non-agitated patients
Stewart & Bowers, 2013 [54]	Only incidents of racism are predicted by age: OR = 1.04 (95% CI = 1.01–1.07) p = .008
Biancosino et al., 2009 [65]	Patients in hostile and violent group were younger: χ^2^ = 20.35; p = .026
Carr et al., 2008 [66]	Being >25 years of age: AOR = 0.23–0.59
Ketelsen et al., 2007 [69]	Age 42.1 years (SD = 15.6) in the group of aggressive patients: T = 4.1; p *<* .001
	OR = 1.07 (95% CI = 1.04–1.11) p *<* .001
Abderhalden et al., 2007 [72]	Age under 29: OR = 1.04 (95% CI = 0.75–1.43) p = .82
	Age over 49: OR = 0.67 (95% CI = 0.46–0.99) p = .04
Raja & Azzoni, 2005 [102]	Violent patients are younger: F = 6.02; df 2341; p = .002
Krakowski & Czobor, 2004 [110]	Violent patients 33 years younger (*SD* = 8.2) vs 36.2 (*SD* = 8.5)
Chang & Lee, 2004 [78]	Younger age: t = -2.44; df = 109; p = .02
Bowers & Alexander, 2003 [119]	Greater age had higher frequency of events
Grassi et al., 2001 [87]	Violent patients were younger: t = 4.07; p *<* .01

First colum is first author and year of publication and reference, OR: Odds Ratio, CI: 95% Confidence Interval, t: t-score, χ^2^: Chi-square, df: degrees of freedom, F: ANOVA test statistic, SD: Standard Deviation.

#### History of violence

In 25 studies, an association between the development of aggression and a history of violence was examined; the average score on the OSQE was 6.9 (SD = 1.9 range 3–11) [43, 48, 50, 54, 55, 58, 60, 66, 67, 73–75, 86, 88, 90, 91, 94, 104, 107, 115–118, 120, 121].

Violence in a previous admission proved predictive for aggression in a subsequent admission (OR = 1.34; 95% CI = 1.12–1.93) [115, 117]. Within the same admission, a less serious incident increased the risk 5 times for a more serious incident later on [66].

A history of aggression was found to have ORs between 1.45–7.25 for aggression during the admission [43, 48, 54, 55, 74, 75, 88, 104]. In 3 studies, a history of violence did not predict current aggression [50, 67, 90]. Some studies only made narrative comments. For example, researchers stated witnessing abuse earlier in life and having a legal history of assault conviction were predictive for aggression on the ward [118]. Furthermore, interviewed nurses proclaimed that a history of violence was associated with the occurrence of aggression on the ward [120]. Higher rates of conflict were found when a higher proportion of patients was admitted for being at risk of harming others [121].

Overall, a history of aggression seems to be a factor contributing to the development of aggression on the psychiatric ward.

#### Substance abuse

In 22 studies, a correlation between substance abuse and the occurrence of aggression was reported [42, 49, 50, 54, 58, 65, 73, 75, 88, 91, 92, 97, 98, 101, 106, 111, 114, 117, 120, 122, 123]. The average score on the OSQE was 7.2 (SD = 2.4; range 2–12).

Severely agitated patients were found to use substances more often compared to non-agitated patients: 7.1% vs 13.5% (χ^2^ = 9.07; p *<* .005) [106]. The risk of aggression was enlarged by current use of alcohol (OR = 2.19; 95% CI = 1.30–3.70; p = .003) and drug abuse (OR = 2.60; 95% CI = 1.54–4.37; p = 0.0) [111]. When there was a history or diagnosis of substance abuse more verbal and physical aggression towards objects and others was found (Spearman correlation (r) = 0.19; *p <* .01) [58] and it enlarged the risk for aggression: OR = 20.10 (95% CI = 5.03–80.27; p *<* .01) [75], OR = 5.47 (95% CI = 1.66–17.96; p = .005) [65], OR = 2.893 (95% CI = 1.21–6.94; p = .017) [50]. For alcohol abuse the OR = 10.37 (p = .03) [88].

Specifically patients with a bipolar disorder and a positive drug screen for cannabis had more episodes of agitation (p = .033) and more use of medication pro re nata (p *=* .008) [123].

One study using a momentary assessment strategy found that physical aggression was associated with alcohol craving (OR = 6.26; 95% CI = 1.82–21.46), cigarette craving (OR = 8.02; 95% CI = 1.67–38.43) and symptoms of withdrawal (OR = 11.05; 95% CI = 3.16–38.65) [49]. Nurses considered the use of drugs or alcohol as an inducement to aggression [42, 120].

Finally, 4 studies failed to show an association between substance abuse and aggression development [92, 97, 101, 114].

Overall it can be concluded that current, or a history of substance or alcohol abuse is indeed a factor influencing the occurrence of aggression on the inpatient ward.

#### Insight

Five studies focussed on illness insight in relation to aggression development [65, 94, 108, 109, 116]. No association for illness insight and aggression development was found in a cohort of 29 patients with schizophrenic disorder or bipolar disorder who completed the Insight Scale [116]. The PANSS scores for lack of insight, where aggressive patients score higher (5.1) compared with non-aggressive patients (4.3). On the BPRS the amount of insight is scored, where aggressive patients scored lower (4.4; p < .001) compared to non-aggressive patients (6.11), with χ^2^ = 32.44; p *<* .001 [109]. Violent patients of a cohort of 64 inpatients with a diagnosis of schizophrenia or schizoaffective disorder showed reduced insight in their delusions (z = -3.26; p *<* .01) [94]. No conclusions can be drawn whether insight of the patient into his illness is a clear contributing factor to the occurrence of aggression.

#### Other factors

Patient related factors that could not be classified in the aforementioned factors are briefly summarized in this paragraph. Aggressive patients may on average be admitted longer on wards (24.9 days vs 12.1 days with F = 68.34; p < .001 and 14 days vs 7 days (without further statistics)) [73, 91].

Aggressive patients more frequently used less antipsychotic medication on admission: 38.8% vs 76% (χ^2 =^ 9.07; p *<* .05) [106]. In the first night after admission a short sleep duration correlated with higher scores on the Brøset Violence Checklist the following day, irrespective of psychiatric diagnosis (r = -0.38; p = .01) [124]. Living alone (OR = 0.55; 95% CI = -0.31– -0.96), being unemployed, or retired (OR = -0.74; 95% CI = 0.57–0.97) was associated with more incidents of aggression [48]. Patients declared that external stressors, such as financial worries, only increased stress and agitation inducing the development of aggression [125]. When patients were non-compliant to treatment the risk for aggression increased (OR = 1.14; 95% CI = 1.00–1.35) [115]. A first admission showed a decreased risk for aggression compared to concurrent admissions (OR = 0.52; 95% CI = 0.29–0.93) [54]. Compared to non-aggressive patients, aggressive patients were more likely to have interpersonal problems (OR = 5.53; p *<* .001), have been in foster care as a child (OR = 10.87; p *<* .001), have migrated from their country of origin (OR = 3.50; p *<* .04), have divorced parents (OR = 5.38; p *<* .001) or have a parent or sibling with psychiatric problems (OR = 5.49; p = .01; no confidence intervals provided) [55].

Patients who were involuntarily admitted showed more aggression (χ^2^ = 4.38; p = .04 [59], OR = 3.32; 95% CI = 2.11–5.24 [69], OR = 2.16; 95% CI = 1.63–2.9 [72], OR = 10.96; 95% CI = 2.35–51.02 [79], OR = 1.56l 95% CI = 1.32–1.85 [48], and χ^2^ = 324.412; p < .001 [102]). However, other studies were not able to determine any statistical significant contribution of involuntary admission [62, 74]. A higher ratio of involuntarily admitted patients on the ward is reflected in more aggression [121].

One study found that patients with akathisia show more threatening behaviour (F = 4.88; p = .031) and more physical aggression (F = 6.15; p = .016) [126].

### Staff factors associated with aggression

Fifty-five studies reported on staff factors that may be associated with aggression at psychiatric wards, including the quality of interaction between staff and patients. The average OSQE-score was 4.9 (SD = 2.0; range 2–9) [28, 29, 42, 43, 45, 64, 100, 103, 104, 120, 121, 127–151]. This is remarkably lower than the average score of the articles published on patient factors. This is due to a lack of inclusion and exclusion criteria and a methodology with mainly interviewing staff, which results in a poor score for assessment validity for both the dependent as independent variables.

#### Gender

Fourteen studies reported data on the gender of the nurse in relation to aggression development, depicted in Table 5 [28, 29, 103, 104, 120, 127–130, 133, 138, 139, 141, 142, 144, 145, 150, 151]. In 5 studies, no gender differences were found [28, 104, 120, 129, 130], whereas 9 studies reported that male nurses encountered more aggression (see Table 5), and no clear methodological difference was found on the OSQE. One study reported that female nurses met more aggression [141], but this study was of poor methodological quality with an OSQE score of 3. Three studies concluded that aggression was more frequent between patient and staff of the same gender (r = 0.32; p < .001), with medium methodological quality (OSQE score 5, 6 and 6).

**Table 5:** Included studies on the association of gender of staff and aggression development in patients.

Author	How gender is associated with the development of aggression
Pekurinen et al., 2019 [127]	Male vs female: OR = 1.90 (95% CI = 1.23–2.96) p = .004
Ezeobele et al., 2019 [128]	Being male (nurse) enlarges risk for aggression: z = -4.82 (p *<* .001)
Yang et al., 2018 [29]	Male nurse experienced higher incidence of incidents in the past year: z = -3.76 (p *<* .001)
Zeng et al., 2013 [133]	Male nurse: OR = 2.5 (95% CI = 1.1–5.6) p = .02
Flannery et al., 2011 [139]	Male nurses were more likely to experience physical aggression (92%) and female nurses were more likely to experience verbal threats (7%). χ^2^(5) = 37.04 (p *<* .0001)
Virtanen et al., 2011 [138]	Male nurse: OR = 1.24 (95% CI = 0.93–1.65)
Chen et al., 2009 [141]	Physical violence: being a female nurse: ARR = 4.48 (95% CI = 2.53–7.92)
	Verbal aggression: being a female nurse: ARR = 15.64 (95% CI = 9.21–26.84)
Bowers, 2009 [142]	35% of male nurses experienced aggression: r = 0.221 (p = .01)
Knowles & Brown, 2008 [144]	Physical aggression: 43.8% took place on the male patient ward with 64.3% against male nurses
	56.3% took place on the female patient ward with 70.4% against female nurses
	Verbal aggression: 49.7% took place on the male patient ward with 39% against male nurses
	50.3% took place on the female patient ward with 74.7% against female nurses
	This difference remained significant after controlling for staffing ratios: aggression against same sex is more common
Flannery et al., 2007 [145]	Same-gender assaults are statistically significantly more frequent: χ^2^ (1, N = 1551) = 157.45 (p *<* .0001)
Hamadeh et al., 2003 [150]	Assault rates are highest in males: RR = 1.81 (95% CI = 1.00–3.25)
Omerov et al., 2002 [103]	Of the male patients, 67% was violent against female nurses and 33% to male nurses. Of the female patients, 93% was violent to female staff.
	Association between gender of nurses and patient is r = 0.32 (p *<* .001)
Shepherd & Lavender, 1999 [151]	34% of male nurses was victim of violence and 7% of female staff: significant difference. χ^2^ = 8.56; df = 1 (p *<* .01)

First colum is first author and year of publication and reference, OR: Odds Ratio, CI: 95% Confidence Interval, χ^2^: Chi-square, df: degrees of freedom, r: correlation coefficient, ARR: Adjusted Risk Ratio, RR: Risk Ratio.

With limited evidence, it appears that male nurses were more often involved in aggressive incidents, which does not mean that they contribute to the occurrence of aggression because of their gender.

#### Age

Focussing on the age of staff members, younger nurses were more at risk to experience aggression against them (OR = 0.96; 95% CI = 0.94–0.98) [127]; an age under 30 years has an Adjusted Rate Ratio (ARR) = 1.70 (95% CI = 1.17–2.48) for physical aggression [141] and the age group 30–39 years has an OR = 1.04 (95% CI = 0.69–1.57) [138]. Interestingly, staff with an age between 40–49 years and 50–63 years both experienced less aggression, respectively an OR = 0.66; 95% CI = 0.44–1.01 and an OR = 0.54; 95% CI = 0.34–0.86) [138]. Eight studies failed to show significant associations of which four scored poor on the OSQE (scores 4 and 5) and four scored better (scores ranged 6–8) [29, 120, 129, 130, 133, 140, 142]. Age is not a clear discriminating factor.

#### Work experience

In 13 studies, the years of professional experience or education level of nurses was studied as a potential factor associated with the occurrence of aggression on the ward [28, 29, 43, 104, 120, 128–130, 133, 140–142, 146]. Five studies did not find any significant association [28, 29, 129, 130, 142]. In the remaining studies, the results are contradictory; increased risk for aggression or assault was found with more years of experience (z = 3.05; p *<* .002) [128] or OR = 0.95 (95% CI not provided; p = .001) [120], as well as with fewer years of experience (ARR = 1.23; 95% CI = 1.32–1.18 [141] and ARR = 3.08; 95% CI = 1.47–6.44 [140]). The contradictory results can not be explained by methodological differences, while both high and low scores on the OSQE were present for the different outcomes. Staff members with academic education levels experienced more aggression in one study (OR = 3.0; 95% CI = 1.03–8.9) [133], but not in another [140]. Staff training in aggression management showed no effect on the number of aggressive incidents [43], but this study was of poor methodological quality (OSQE score of 4) and during the study period many other changes such as a reduction in beds were implemented, which made results difficult to interpret. No clear effect of work experience to the occurrence of aggression was found.

#### Staffing level

Inadequate numbers of staffing led to more aggression according to interviewed nurses [29, 120, 130]. In contrast, 2 studies reported an opposite effect; more staff present was associated with more aggressive incidents on the ward, however not statistically significant [131, 137] and the OSQE score was low (3 and 5). Authors of one of these studies discuss that this result might be due to a bias: when there is more aggression on the ward, more staff is present [131]. Wards with high rates of conflict and high rates of containment were associated with higher levels of unqualified staff (F (df = 3) = 8.84; p *<* .001) and higher levels of temporary staff (F (df = 3) = 6.64; p *<* .001) [121]. Staff being more than average absent from the ward was a positive predictor for incidents (IRR = 1.11; 95% CI = 1.06–1.16) [147].

#### Interaction between nurses and patients

There were 24 studies included discussing the quality of interaction between nurses and patients in relation to aggression development [42, 46, 60, 63, 68, 69, 81, 82, 85, 87, 99, 103, 104, 108, 125, 129, 130, 152–158]. The average score on the OSQE for these studies was 5.2 (SD = 2.4; range 1–10).

Studies that interviewed staff pointed to several factors explaining aggression development. Poor communication between staff and patients [153, 155, 158] and a perceived lack of empathy, respect and distance or a lack of shared decision making [125] led to aggression. Other interactions with patients that contribute to aggression have been clustered here and weighted percentages were calculated for the contribution to the development of aggression as mentioned in different papers. The interaction limit setting (which is the intervention of nurses to explain to the patient which behaviour is not tolerated, e.g. denying cigarettes, telling the patient not to leave the ward) was seen as contributing factor in 21% of the incidents [42, 46, 60, 63, 68, 81, 82, 85, 87, 99, 103, 104, 130, 152, 154, 157, 158]. Communication in general between staff and patient (other than limit setting, e.g. talking to the patient) contributed in 38% of the cases [81, 82, 104, 129, 152–156] and interaction between patients in 29% [42, 63, 85, 87, 104, 108, 130, 153]. Help with Activities of Daily Living in 8% [87, 103, 130] and medication administration in 7% [87, 104, 108, 129, 130, 152, 157].

No provocation or trigger was found in the interaction in 37% [46, 60, 69, 87, 108, 129, 130, 157]. From these results, interaction was an important factor in the occurrence of aggression, especially communication in general between staff and patients.

#### Other staff-related factors

One study on the relation between aggressive incidents and perceived workload or stress experienced by staff, showed that job strain increased the risk for aggression (OR = 1.65;95% CI = 1.07–2.54), assessed in nurses with the Job Content Questionnaire [127]. The risk for aggression was increased with overwork, tiredness and job dissatisfaction [143], as well as a negative staff morale [136], poor collaboration among nurses [45], poor satisfaction with leadership (OR = 1.83; 95% CI = 1.03–3.24) [127], and a lack of good introduction for new nurses [143]. Furthermore aggression was associated with a moderate to high level of anxiety of the nurse (ARR = 2.75; 95% CI = 1.14–6.61) [140] and working in rotation shifts (OR = 1.68; 95% CI = 1.05–2.07 [28] and OR = 4.7; 95% CI = 2.2–9.9 [133]. In contrast, job satisfaction and stress showed no association with experience of violence in the past 12 months [45, 129].

Nurses who believed that aggression by patients is preventable, experienced lower rates of incidents in the past year: z = -2.02; p = .044 [29]. Scores on the Maslach Burnout Inventory were not significantly associated with experiencing aggression, except for the score on depersonalisation with r = 0.276; p = .001 [142].

Nurses stated a lower risk of violence was present when they worked as a team and their personal knowledge was taken into account in the treatment decisions [149]. Less aggression was reported when a team scored better on team functioning, had more positive attitudes to difficult patients, lower burnout and had more order and organization on the ward [121]. Lower aggression rates were also found with a proactive ward manager, positive teamwork, staff feeling less pressured or having lower workload, staffing change with positive impact and staff feeling supported [136].

### Ward factors associated with aggression

37 studies were included discussing how ward factors influence the occurrence of aggression, with an average score on the OSQE of 5.7 (SD = 1.9; range 2–9) [29, 33, 63, 64, 72, 73, 84, 85, 87, 91, 93, 103–105, 112, 120, 125, 130, 132, 136–138, 142, 146, 147, 150–155, 159–164].

#### Bed occupancy rates

Patients as well as staff members reported overcrowding as a potential reason for aggression on the ward [63]. The average bed occupancy when incidents occurred was 77% in one study, opposed to 69% when no incident occurred (χ^2^ = 7.9; df = 1; p = .005) [162]. The net occupancy of beds was higher (98.6% +/-14.8) on days with aggressive incidents compared to days without (95.7%+/-15.7); p *<* .0001 [161]. The larger the total number of patients on the ward, the higher the number of incidents (r = 0.634; p = .05) [84]. A 5% surplus of patients compared to the number of beds enlarged the risk for aggression (OR = 1.30; 95% CI = 0.72–2.34), as well as an excess between 5% and 10% (OR = 2.08; 95% CI = 1.26–3.44) and an excess above 10% showed the largest risk (OR = 2.15; 95% CI = 146–3.167); p *<* .001 [138]. One study found no correlation between the number of patients on the ward and aggressive incidents [93]. The evidence of overcrowding as a contributing factor to the occurrence of aggression is contradictory, but there is some evidence that overcrowding is accociated with the occurrence of aggression.

#### Admission as risk factor

Within a shift in which a new patient was admitted on the ward the nurse was more likely to experience aggression (r = 0.02; p = .002) [137]. Especially, admissions of younger patients (<36 years) increased the risk (IRR = 1.17; 95% CI = 1.01–1.37), as did admissions of males in the first week after admission (IRR = 1.17; 95% CI = 1.01–1.35) [147].

#### Location

Seven studies reported on the location of incidents in a hospital [29, 85, 87, 104, 112, 150, 151]. Most incidents happened on acute admission or locked wards with a prevalence ranging from 44%–62%, on chronic wards the prevalence was 30%, in the outpatient department 4.5% and during home visits 3% [29, 150]. In contrast, one study reported more incidents on a low secure unit compared to a medium secure unit (χ^2^ = 21.45; df1; p *<* .001).

Aggressive incidents were reported in hallways (14%–67%), the day or activity room (13%–28%), patient bedroom (8.5%–21.9%), dining room (7%– 17%), staff office (6.1–19%) and in the courtyard of the unit 3.3% [85, 87, 104, 112, 138].

#### Time

In 18 studies, the time of incidents was compared (see Table 6) [29, 72, 73, 85, 87, 91, 104, 105, 112, 120, 130, 137, 146, 147, 150, 161–163], showing no clear distribution within the time of day. The time of day when the psychiatrist and nurses walked rounds resulted in more incidents (27%) [29].

**Table 6:** Included studies on the potential association of time of day and aggression development.

Author	Time as a ward factor
Yang et al., 2018 [29]	Of all the incidents:
	54.3% during the day shift
Giarelli et al., 2018 [112]	Of all the incidents:
	57% during the day shift
	40% during the evening shift
Ewing & Castle, 2018 [105]	Of all the incidents:
	38.3% in the morning
	39.4% in the afternoon
	22.3% in the night
Sato et al., 2017 [130]	Of all the incidents:
	Between 9.00–16.59: 49.6%
	Between 17.00–24.59: 31.5%
	Between 01.00–8.59: 17.5%
Al-Azzam et al., 2017 [120]	Of all the incidents:
	Day shift: 20%
	Evening shift: 7%
	Night shift: 2%
	Not specified: 39%
Teitelbaum et al., 2016 [161]	Of all the incidents:
	44% in the morning shift
	41% in the afternoon shift
	15% in the night shift
Bowers, 2012 [137]	Highest rate of conflict in morning shifts: mean 4.88 incidents (mean in the evening 3.69): χ^2^ = 164.31; df = 2, p *<* .001
	Highest rate on Wednesday (mean 4.75), lowest on Sunday (mean 4.05): χ^2^ = 25.13; df = 6; p *<* .001
Bowers et al., 2007 [146]	All incidents together: less likely to appear in weekends: χ^2^ = 10.96; df-1; p = .001
	No significant difference for physical aggression during days of the week
	No significant difference for days on which ward round took place
Bowers et al., 2007 [147]	Winter season: more incidents: adj r2 = 0.059; p *<* .0005
Abderhalden et al., 2007 [72]	Peak of incidents between 10–11 am and 5–8 pm
	Equally distributed over days of the week, except for Sundays: less incidents
El Bari & Mellsop, 2006 [73]	52% in evening shift
	27% in day shift
	21% in night shift
Hamadeh et al., 2003 [150]	56.7% in the evening
	16.4% in May (highest percentage), only 3% in February and December (lowest percentage)
Chou et al., 2002 [104]	44.6% in the day shift
	46.7% in the evening shift
	8.7% in the night shift
Nijman, 2002 [6]	32% of outwardly directed aggression took place between 6 and 12 pm and 7% between 8 and 9 pm
Ng et al., 2001 [162]	Of the 33 physical incidents 32 happened in the morning and afternoon shiftp
	Shift time was related to whether or not an incident occurred: χ^2^ = 12.3; df = 2; p = .002
Manfredini et al., 2001 [163]	Of all the incidents:
	4.5% between 0.00–5.59
	32.6% between 6.00–11.59
	39.5% between 12.00–17.59
	23.4% between 18.00–23.59 p *<* .001)
Grassi et al., 2001 [87]	Of all the incidents:
	45.6% between 8.00–13.00
	35.9% between 14.00–20.00
	18.5% between 21.00–7.00
Barlow et al., 2000 [91]	49% between 7.00–15.30
	36% between 15.30–22.30
	15% between 22.30–7.00

First colum is first author and year of publication and reference, χ^2^: Chi-square, df: degrees of freedom, r: correlation coefficient.

#### Climate of the ward, restrictions

Patients reported that contributing factors to aggression were: unhygienic surroundings and not being heard when complaining about this, the quality and quantity of food, the unavailability of daily necessities, the lack of privacy and the realization that personal information was not handled confidential, noise levels on the ward, crowding or the place felt prison-like, a negative experience of seclusion, unfair limit-setting and poor communication about this, and a lack of structured activities [132].

Staff members thought that problems with legislation and the way hospital management dealt with aggressive incidents contributed to the occurrence of aggression [120]. Behavioural restrictions belonging to operational procedures and house rules, such as restrictions on personal possessions (phone, scheduled showers), reduced access to familiar coping strategies (smoking, listening to music), boredom and physical confinement (feeling as loss of autonomy) led to more aggressive incidents [63, 125, 153–155]. This worsened when policy was not executed consistently [125].

Feeling unsafe in the ward led to angry outbursts and disruptive behaviour [132]. Noise on the ward was associated with patients having more thoughts about violence (OR = 2,82; 95% CI = 1.42–2.81) [49], which might have led to more aggression.

When staff approached a patient with overpowering numbers, applied manual restraint or locked the doors of the ward for 1–3 hours, more aggressive incidents took place [64]. In an orderly, predictable structured environment [136, 142] less aggressive incidents took place.

Patients stated that smoking habits led to aggression (e.g. because smokers and non-smokers shared rooms and established smoking restrictions) [132]. A smoking ban showed a 39% reduction in the number of violent assaults per month (Incidence Rate Ratio (IRR) = 0.61; 95% CI = 0.53–0.70) and a 47% reduction in patient to staff assaults (IRR = 0.65; 95% CI = 0.44–0.63) [160].

Different restrictions and atmospheric factors on the ward seem to influence the occurrence of aggression on the inpatient ward, offering possibilities for change on the ward itself.

## Discussion

The aim of this systematic review was to provide an overview of the scientific literature addressing the prevalence of, and factors associated with aggression on psychiatric wards. In accordance with previous studies, contributing factors are subdivided into patient, staff and ward-related factors [5, 165].

### Prevalence of aggression

Aggression is a serious issue in mental health care, where safety of both patients and staff needs to be protected. The weighted mean prevalence of aggressive events was 54%, with a wide prevalence range between studies (7.5% to 75.9%). This prevalence range deviates from a previous review, reporting a 3–44% range [18]. An explanation for this discrepancy is the conceptualization of aggression used in this review. Applying a broad interpretation of the definition of aggression: a continuum of *agitation–aggression—violence* resulting in a larger sample of studies which may reflect in the larger prevalence interval.

Only a small proportion of the admitted patients is responsible for the majority of the aggressive incidents: 0.5–33% of patients cause 42–66% of the incidents. The first three days of admission appear to be related with the highest prevalence rates, which is in accordance with an earlier review by Woods and colleagues (2007) [166]. This finding may be explained by several factors, such as symptom severity, intoxication at the moment of admission, admission without consent or understanding, or the patient having to wait for hours, being tired or hungry.

The prevalence of experiencing aggression among nurses in mental health care is 25%-80%. This is in line with an earlier review, which showed that the majority of staff in acute psychiatric units have been assaulted at some point in their career [167].

Although the prevalence of aggression rates vary, they underline the scale and impact of the problem. Programs aimed at reducing aggression should focus their actions on the first days after admission and on helping staff to deal with aggression.

### Development of aggression

#### Patient factors

In the current review identified patient factors that contribute to an increased risk of aggression development are: being diagnosed with a psychosis-spectrum disorder, a personality disorder or a bipolar disorder (especially a manic state). At symptom level, paranoid delusions, impulsivity and hostility increase the risk for aggression development. These findings are a replication of previously published reviews on patient factors associated with aggression [5, 7–10, 20, 22–24, 166, 168–170]. The individual studies included in these reviews have largerly been excluded from the current review because of a date before 1999 [8, 22, 23, 166, 168, 169], or because they were on outpatient clinics [20]. Therefore, this current review contains an update on the relation between diagnosis and the occurrence of aggression on the inpatient ward.

A diagnosis of major depression or higher scores on feeling down or gloomy at symptom level decreased the risk of aggression. An earlier review by Dack (2013) found this decrease in risk for aggression for depression, bipolar disorder, adjustment disorder and substance abuse [8]. In the present review, hallucinations were not found to be associated with aggression development, whereas in a previously published review it was concluded there is contradictory evidence: both significant associations as well as no association between hallucinations and aggression were found [19], but this review was conducted with studies before 1999.

A minority of the studies (n = 13) included here confirmed male gender as a factor influencing the occurrence of aggression, but most studies found no relation (n = 22). Previous reviews concluded the same [8, 10, 145, 166], but other previous reviews concluded that the male gender indeed is a risk factor [9, 25]. Other studies concluded aggression occurred mainly between same sex victim and aggressor [7, 22]. It remains unclear how these differences can be explained and therefore the conclusion is that a male gender might be a risk factor. But this still needs to be studied further.

The review by Dack and colleagues (2013) concluded that younger age is a risk factor for aggression [8], which was reported before by Aquilina, concluding that mainly patients under the age of 40 were aggressive [22]. However, this is not supported in the present review. Only a minority of studies reported a young age as a risk factor and older age as a protective factor. This is in line with the review of Cornaggia who concluded that there is only weak evidence for younger age as a risk factor [7].

In spite of the use of different assessment methods, all studies indicated that a history of aggression (broadly defined) predicted renewed occurrence of aggression. This is in line with earlier reviews where a history of aggression was significantly associated with aggression in the current admission [8, 9, 23, 25, 166, 171, 172].

Substance abuse may be a factor contributing to aggression, since a majority of studies (18 out of 22) concluded some attributing effect. This is in line with the reviews written in the past [8, 23, 25, 166]. An additional conclusion from this current review is that craving for alcohol or cigarettes and withdrawal symptoms were found to be significant factors in the occurrence of aggression.

Although homelessness and insight have been mentioned as a risk factor in a review by Witt in 2018 [9], only four (2.7%) studies were found examining these factors. From the present review no clear conclusions can be drawn about the association with aggression of these factors.

In the review by Dack and colleagues (2013), involuntary admission was found to be a contributing factor [8], which was also found in this current review in 6 studies. However, higher rates of involuntary admitted patients are also associated with more aggression on the ward. Whether this result is causal or only part of a multifactorial increase of risk cannot be concluded from the data, as this is not studied as such. But involuntary admission appears more likely in patients with aggressive behaviour, as losing autonomy and freedom urges people to resist and become aggressive as means to regain freedom. This is confirmed in earlier work, where it was stated that a locked door seems to frustrate patients [173] which might lead to aggression.

Clinical variables such as more severe symptoms and unresponsiveness to treatment have shown to be better predictors of aggression than demographic variables [23] and these can be supported by several assessment instruments of which the Brøset Violence Checklist (BVC) and the Violence risk 10 (V-RISK-10) were reviewed as the most feasible to be used in acute mental health care settings [174]. This prediction is typically used when a patient is agitated so that a prediction is made whether the patient will become aggressive. Nevertheless, when aggression is seen as a consequence of interplay between patient, staff and ward factors the prediction of aggression is multifactorial and attention for only patient factors seems rather incomplete. Keeping in mind that some risk factors are dynamic and interactive in the current ward situation, whereas others are stable immutable constructs (for example previous hospitalisations or a history of violence). Treating the patient relieves some of the clinical variables, but aggression is seen mostly in the first few days of admission in which treatment is not yet effective. Therefore staff and ward factors are more suitable for preventing aggression on the ward.

#### Staff factors

Staff on psychiatric wards are not only victim to aggression, but also contribute to the occurrence of aggression since aggression is interpersonal. Aggression development in patients is positively related to male gender of staff, job strain, job dissatisfaction, overwork, dissatisfaction with leadership, tiredness, lack of good introduction of the nurse, poor collaboration between nurses, more temporary staff, and more anxiety in nurses. This is a replication of the findings in earlier reviews, but none of them integrated these factors in one review [10, 16, 21–23, 172]. Often these factors have been addressed individually without considering the interplay between them and they have not been systematically replicated in many studies. Therefore, the strength of these findings is questionable, and the clinical relevance needs to be interpreted with caution. Results on the staff factors age and staffing level were inconclusive [10, 16, 22, 175].

Some form of interaction between patient and staff precipitates aggression in 40% of the incidents [5, 10, 16, 25, 119, 166, 176]. These interactions are poor communication, lack of empathy or respect, lack of shared decision making, restrictions for patients, limit setting, patients being denied something, help with ADL, serving medication or discussion about medication, interaction with other patients and discussion about cigarettes. Since this is a finding which has been replicated in earlier reviews including mainly papers before 1999 this is a firm conclusion, although it is yet unclear why exactly this correlation exists while prospective research is missing.

Future studies should address explicitely patient-staff interaction and factors as job strain, job dissatisfaction and overwork that influence this interaction. These factors are crucial for a safe atmosphere on wards and essential for management in guiding ward staff appropriately.

#### Ward factors

The ward in itself may also contribute to more aggression development, which was also concluded by Welsh in their review on environmental factors [21] regarding aggression development. Higher patient occupancy rates and more admissions (specifically of male admissions under the age of 36) are most undisputed factors from this current review and are mentioned in earlier reviews as well [5, 10, 16, 21, 23, 25]. Furthermore, busy places, such as corridors and living areas, with more intense patient to patient and patient to staff interaction, seem more prone for aggression to take place, which was also found in earlier reviews [5, 10, 16, 23].

Numerous other factors have been examined, such as the perceived (absence of) privacy for patients, personal space and freedom to move around, restrictions placed upon patients, inconsistent following of the rules, feeling unsafe, noise on the ward and the feeling of physical confinement. All these are mentioned in earlier reviews [5, 10, 23]. The current review adds to this evidence in that the earlier reviews included mainly studies before 1999 and also included studies on prediction or the management of aggression. The evidence suggests an association between these factors and the occurrence of aggression on the psychiatric ward, but research is only descriptive, thus further research is needed to be able to draw definitive conclusions.

The locked doors of a closed ward may be perceived by the patient as being controlled, although it may also provide a secure feeling. For staff it reduces the need for close observation and protects the ward from the outside [173, 177, 178]. Previous reviews were not in agreement whether locking the doors leads to more aggression [5, 173] and also in this present review it could not be confirmed that a locked door of the ward leads to more aggression.

The model of High Intensive Care (HIC) has been adopted in many psychiatric institutions in Europe. The HIC model focusses on creating an optimal healing environment, with as much autonomy and privacy as possible. Preventing unnecessary escalation is persued through a welcoming atmosphere, frequent contact between patients and nurses that focus on the strengths of a patient, frequent contact with ambulatory carers, multidisciplinary staff, minimum use of coercive measures, and in case of agitated behaviour or aggression the possibility of one-on-one nursing [179]. The results of this review underline the importance and feasibility of making these alterations in ward atmosphere and procedures that may help preventing aggression development on the inpatient ward. More explicitly the findings of this review are important for both clinicians and managers of inpatient psychiatric wards and may offer useful information to make improvements in their ward or staff in order to prevent aggression development on their wards. Staff needs to be taken good care of by good leadership to prevent job dissatisfaction, job strain or tiredness due to overwork. New nurses need a clear introduction program set out for them and the use of temporary staff should be limited. Staff needs sufficient training in communication skills in order to be able to communicate with respect and empathy and let the patient share in the decision making as much as possible. It is important to avoid patronizing communication with a patient. On the ward, occupancy rates should never exceed the number of beds, patients need enough space to roam about the ward and crowding on the ward should be avoided as much as possible, given the higher risk with aggression development. Patients should also have enough privacy. Restrictions and rules should be limited to the absolute minimum and if necessary, the limitations need to be executed consistently by all staff members. In the first days following admission aggression is most likely to take place. Therefore, staff needs to keep an eye on newly admitted patients more closely during the first few days for signs of agitation and intervene as early as possible in the continuum of agitation–aggression–violence.

## Limitations

The heterogeneity of the studies in this systematic review is large because of differences in quality of methodology and a range of definitions of aggression in the included studies. The variability of the concept aggression in the literature is large with the majority of articles not providing a clear definition or comment on which definition was used. Mostly, agitation, aggression and violence are recognized as stages of aggression and used interchangeably, with indistinct and unclear underlying concepts and demarcations. However, also incidents or assaults are studied without a clear definition. Comparison of outcome for the different stages of agitation-aggression-violence was not feasible because of these poorly described definitions. That is why in the present review all these different definitions and concepts were taken together. It would be advisable that studies define the level of aggression on the continuum of agitation-aggression-violence.

A range for the search was chosen for 1999–2019, which makes this review a complete overview of the literature of 20 years. Papers written before 1999 were described in older studies and reviews and were most likely of poor methodological quality (based on reading older reviews and their comments on quality) and hospital care was organized very differently [18]. These studies would therefore not have added additional information to the already large amount of data supplied here. Only papers available in the English language have been included in this review. This is a standard procedure. Therefore, potential important information could have been missed. We chose for only English papers to avoid selectivity based upon our own language availability, which would have led to selection bias. Translation tools enabling researchers to read multiple languages are promising. However, we believe translation tools are still not good enough to be used for scientific literature.

Variations in hospital settings are difficult to compare [18] if described at all. The culture and structure of the wards are unclear, there is a mix of diagnoses, setting (acute admission wards, open vs. closed admission wards, rehabilitation wards), number of beds, patient-staff ratio, bed occupancy rates, and duration of admittance all varying according to national or local standards. This variation in ward setting and local cultures and policies contributes to the wide variation in the outcome. This is unavoidable. An option for controlling for this bias might be stratifying for setting (open versus closed ward), diagnosis and other factors. Given the enormous variation this would have led to very small sub-groups or multiple group comparisons (closed vs open ward, various diagnoses, etc.). It is questionable whether this would have solved the problem of heterogeneity and the related understanding of aggression development, given the small groups. Albeit these limitations, conclusions of the present review are still valid. A clinician or manager may extract factors that apply to their given situation, implementing tools to reduce the development of aggression on their ward.

Prevalence rates are difficult to compare between articles, as they employ a variety of definitions for aggression and differ in methodology: incidents per patient, incidents per week or per month, incidents per occupied bed, incidents per week, per unit, per admission, or per hospital. A consensus and clear description of methodology in every study is required for an objective comparing of the data, however missing [145]. In the present review results were compared when feasible, whereby the provided prevalence rates strengthen the importance and underexposed impression on the vastness of the issue.

A wide variety of assessment instruments are used in the literature to measure aggression, for example the Staff Observation Aggression Scale (SOAS), Overt Aggression Scale (sometimes in modified version), Clinical Global Impression scale (CGI), Positive and Negative Symptom Scale-Excited Component (PANSS-EC), and the Social Dysfunction and Aggression Scale (SDAS). Moreover, studies applied a vast amount of questionnaires lacking information on validity and reliability, obtaining quantitative data with multiple interview techniques or self-designed questionnaires, which are not explained in detail. Quantitative questionnaires on aggression are most often answered by staff and are therefor an interpretation of the staff, which might differ from a patients point of view, introducing a potential bias. To overcome this matter, these studies were described separately in the present review.

Inclusion and exclusion criteria are not always well described. Male patients with a psychotic disorder are overrepresented in the studies, IQ of the patient was never a measured factor. Without severity scores of symptoms, missing in the majority of studies, the severity of the psychopathology remains unclear. Presumably, a large bias has been introduced here and aggression prevalences may be even higher as well as different factors may play a more important role, since the most ill patients are very unlikely to sign informed consent due to suspiciousness, but may show the most aggressive behaviour. With keeping this limitation in mind, results are still applicable to the less ill inpatients.

The quality of papers was divers with scores on the OSQE ranging from 1 to 12 points, reflecting papers with very poor quality to very good quality. This shows that there is a great range in methodological quality of included papers. For the patient factors, the majority of papers had a higher OSQE score of 7 or higher (58%), for the staff and ward factors the majority of papers was of poor quality with a score of 6 and lower (77% and 68% respectively). Despite the wide variety in methodological quality, the large range in quality did hardly reflect differences in outcome. In other words: both articles with very poor quality (reflected by an OSQE score of 2 for example) and articles with very good quality (reflected by an OSQE score of 12 for example) presented similar results. Thus, for most results, the discussion of methodological quality to discriminate between conflicting results was not necessary.

Another limitation constitutes the lack of controlled prospective studies (being only 13%). Furthermore, longitudinal or natural cohort studies that follow-up patients during their stay on the ward and take patient, ward, and staff factors into account, are missing [8]. The predominant retrospective research design hampers the possibility to study the dynamics of aggression development [16] and leads to recollection bias. However, the retrospective design is informative on which factors in retrospect might have contributed to the development of aggression. Also, in studies where interviews were used for finding the factors influencing aggression development patient and staff hold different views on how and why aggression developed. Prospective designs are needed in further research to be able to be more conlusive about causative assocations.

Most studies failed to control for important descriptive factors in their analyses (such as the amount of psychotic patients included in the study, the percentage of involuntarily admitted patients, the percentage of patients with substance abuse, whether a ward was locked or not, staff-patient ratio) which may have confounded the interpretation of the results (expressed through odds ratios).

Notwithstanding these shortcomings, the presented results provide a clear perspective on the magnitude of the issue of aggression in mental health care institutions and support the model of the three main factors (patient, staff and ward) in contributing to the risk of aggression.

Future research requires a prospective design, with clear descriptions of patient factors, including IQ. An IQ between 50–84 is a risk factor for the use of coercive measures [180], but has not been studied as direct factor for aggression. Furthermore ward culture and structure, occupancy rates, locked door and staff indicators such as training, staff-to-patient ratio, mode of communicating and interacting with the patient, and ways of interacting with the patient should be prospectively studied. Data collected from these studies may be used to introduce new policies decreasing aggression in various menthal health care settings.

## Conclusion

Aggression is a major issue among psychiatric inpatient units, with both nurses and fellow patients being victimized. Aggression is best defined as part of a continuum of agitation–aggression–violence. Development of aggression is explained by a combination of factors embodied in patients, staff and the ward. The majority of studies focus primarily on the contribution of patient factors in developing aggression, neglecting ward and staff factors that may be more promising targets for interventions to reduce or prevent aggression development on the inpatient psychiatric ward. Future research should focus on prospective naturalistic studies to gain more insight into the dynamics of aggression development in psychiatric wards, specifically the ward and staff factors. This may help identify more precise prevention and intervention strategies.

## Supporting information

S1 Checklist PRISMA 2009 Checklist. (DOC)

S1 File Search strategy. (DOCX)

S2 File The development of a methodological criteria list for observational studies, the Observational Study Quality Evaluation (OSQE). (DOCX)

S1 Table Included studies on prevalence of aggression measured in a cohort of mental health workers. (DOCX)

S2 Table Included studies on prevalence of aggression measured in patient cohorts. (DOCX)

S1 Dataset Dataset review. (XLSX)
