# Supplementary material for: Aggression on the psychiatric ward: Prevalence and risk factors. A systematic review of the literature
Source: PLoS One. 2021 Oct 8;16(10):e0258346. doi: 10.1371/journal.pone.0258346 (PMC8500453; doi:10.1371/journal.pone.0258346)
Supplement: S1 File — (DOCX) [file pone.0258346.s002.docx]

S1 File

Search on Pubmed:

(((((((("aggression"[MeSH Terms] OR "aggression"[All Fields]) OR ("aggression"[MeSH Terms] OR "aggression"[All Fields] OR "aggressive"[All Fields])) OR (("violence"[MeSH Terms] OR "violence"[All Fields]) OR violent[All Fields])) OR (agitation[All Fields] OR agitated[All Fields])) AND (("psychiatry"[MeSH Terms] OR "psychiatry"[All Fields]) OR ("psychiatry"[MeSH Terms] OR "psychiatry"[All Fields] OR "psychiatric"[All Fields]))) AND ((("inpatients"[MeSH Terms] OR "inpatients"[All Fields] OR "inpatient"[All Fields]) OR ("hospitals"[MeSH Terms] OR "hospitals"[All Fields] OR "hospital"[All Fields])) OR ward[All Fields])) NOT (("dementia"[MeSH Terms] OR "dementia"[All Fields]) NOT (("child"[MeSH Terms] OR "child"[All Fields] OR "children"[All Fields]) OR ("child"[MeSH Terms] OR "child"[All Fields]))) NOT (("adolescent"[MeSH Terms] OR "adolescent"[All Fields]) OR ("adolescent"[MeSH Terms] OR "adolescent"[All Fields] OR "adolescents"[All Fields]))

Search Embase

(aggression or aggressive or violence or violent or agitation or agitated) AND (psychiatry or psychiatric) AND (inpatient or inpatients or hospital or ward) AND (not dementia not child* not adolesce*) AND (not forensic not prison not offender) limit to (full text and human and english language and yr="1999 - 2019" and article and journal)
