# Supplementary material for: Aggression on the psychiatric ward: Prevalence and risk factors. A systematic review of the literature
Source: PLoS One. 2021 Oct 8;16(10):e0258346. doi: 10.1371/journal.pone.0258346 (PMC8500453; doi:10.1371/journal.pone.0258346)
Supplement: S2 File — (DOCX) [file pone.0258346.s003.docx]

The development of a methodological quality criteria list for observational studies, the Observational Study Quality Evaluation (OSQE)

**Marjan Drukker^1*^, Irene Weltens^1,2^, Carmen F. M. van Hooijdonk^1,3^, Emma Vandenberk^1^, Maarten Bak^1,2^**

1. Maastricht University, School for Mental Health and Neuroscience (MHeNS), Maastricht, The Netherlands.

2. Mondriaan, Maastricht, The Netherlands

3. Rivierduinen, Institute for Mental Health Care, Leiden, The Netherlands

**Correspondence:**

**Marjan Drukker**

Maastricht University, School for Mental Health and Neuroscience

Maastricht, P.O. Box 616, 6200 MD Maastricht, The Netherlands

Email: [marjan.drukker@maastrichtuniversity.nl](mailto:marjan.drukker@maastrichtuniversity.nl)

Tel: +31 43 388 3913

**Key words:** observational studies, risk of bias, methodological quality criteria list, cohort, case-control study, cross-sectional study

Word count: 4707

Number of tables: 3

Number of figures: 2

**Abstract**

**Background.** Existing study quality and risk of bias lists for observational studies have important disadvantages. For this reason, a comprehensive widely applicable quality assessment tool for observational studies was developed.

**Methods.** Criteria from three quality lists were merged into a new quality assessment tool: the Observational Study Quality Evaluation (OSQE). The OSQE consists of a cohort, case-control, and cross-sectional version.

**Results.** The OSQE cohort, the OSQE case-control and the OSQE cross-sectional include all items applicable to that type of study, for example the representativeness of the study population, the validity of the independent and dependent variables, and the used statistical methods. Before scoring the OSQE, the rater is asked to define how to score items, in detail. A study can obtain a star for each item. Each item also has a veto cell. This cell can be checked when poor quality with respect to that specific item results in a low quality despite stars on other items. Although stars add to a sum score, the comments field is the most important part of the OSQE.

**Conclusions.** The OSQE presented in the current paper provides a short, comprehensive and widely applicable list to assess study quality and therewith risk of bias.

1. **Introduction**

In medicine, psychology or health sciences, when performing a meta-analysis or systematic review, judgement of the methodological quality of the included studies is essential. For randomized controlled trials (RCTs) (1; 2) and systematic reviews (3; 4), various quality criteria lists are available. Criteria lists for observational studies are also available (5; 6; 7; 8), but they have disadvantages. The most recent, the Risk Of Bias In Non-Randomized Studies - of Interventions (ROBINS-I), is extensive and is based on analogy with RCTs. This makes the instrument more difficult to score and not suitable for all observational studies (9). Accordingly, besides the ROBINS-I, a new shorter, intuitively understandable and more comprehensive quality assessment tool to compare quality of observational studies is needed.

The increase in number of published studies in the last decades exceeds the ability of researchers and clinicians to keep track of all the new expanding information. That other authors perform systematic reviews and meta-analyses to summarize findings of individual studies is valuable in acquiring and sharing knowledge. With respect to the hierarchy of level of evidence, RCTs are at the top and, thus, this study design is seen as the gold standard (10). Internal validity is high, confounders are avoided by pre-stratification, randomization and additional methods to create equal groups and intention-to-treat analysis is performed (1; 10). However, RCTs are not always appropriate, adequate or possible (10; 11). To increase internal validity inclusion and exclusion criteria of an RCT usually are so strict that results are valid for a homogeneous sub group of patients only (10). Results could be extrapolated to other patient groups such as patients with comorbidities, drug use, different age groups etcetera, but it can be doubted whether this is valid. In addition, randomization is not always ethical. Risk factors such as exposure to for example asbestos cannot be studied in an RCT, neither can patients be forced to refrain from regular treatment when this treatment is proven effective. Furthermore, because sample size in RCTs is usually low and follow-up is short, rare side effects can only be detected after introduction of a new drug by performing observational studies (12). Finally, the number of hypothesized risk and protective factors for a wide variety of diseases and symptomatology is increasing. By first performing observational studies, researchers can identify which factors are most promising to study in an RCT. Thus, observational studies give additional information next to results from RCTs. Thus, when systematic reviews and meta-analyses are performed to integrate results of individual studies, they should also include observational studies. Observational study designs need their own criteria lists to assess methodological quality.

Within the group of observational study designs, the three most important are cohort study, case-control study and cross-sectional study. A cohort study assesses risk factors in a group of subjects at baseline and follows this cohort over time to assess the outcome (usually incidence of a disease or mortality). A case-control study selects a group of subjects with an illness (cases) and matches these with healthy controls. Subsequently, risk factors are assessed in both groups retrospectively, in order to analyze what risk factors are associated with the case-control status. A cross-sectional study assesses risk factors and the outcome at the same moment in time. This type of study design can be used to assess associations (e.g. exposure to specific risk factors may correlate with particular outcomes). However, making causal inferences is impossible. More details on epidemiological study designs can be found in epidemiological textbooks (such as Rothman 2018 (13)). This vast amount of potential articles holding valuable information for systematic reviews and meta-analyses needs assessment of methodological quality. Previously, various terminology has been used. Synonyms such as “methodological validity”, “study quality” and “methodological quality” have been fashionable at the end of the 20^th^ century and the beginning of the 21^st^ century. Currently, the term "risk of bias" is the standard term used by Cochrane (2). In fact, methodological validity, study quality and risk of bias are very similar concepts. In the present paper, the term methodological quality is used for this construct. When referring to observational studies, some scientists (e.g. (9; 14)) use the term non-randomized studies (NRS). However, NRS also include case studies and case reports. The present paper addresses study quality of cohort, case-control and cross-sectional studies, only. Case studies and case reports have a different criteria list (15; 16). In the present paper, the term “observational studies” includes cohort, case-control and cross-sectional studies, while the term “NRS” is an umbrella term for observational studies, case series and case reports.

Contemporary with the present paper, two systematic reviews were performed for which a suitable methodological quality list was needed. The first systematic review was assessing factors influencing the development of aggression in psychiatric inpatient units (Anonymous, submitted). A search was performed to find studies analyzing factors important for the development of aggression on the inpatient ward, divided in patient, staff and ward factors. The search yielded mainly cohort and case-control studies. The second systematic review assessed dopamine functioning in populations with an increased risk of developing psychosis (Anonymous, in prep). The search contained studies that investigated different parts of the dopaminergic system in high-risk populations and yielded mainly case-control and cross-sectional studies.

While scientific researchers perform systematic reviews and meta-analyses, medical and paramedical students and residents learn to make a “Critical Appraisal of a Topic” (CAT)(1). A CAT is almost similar to a systematic review, but has certain specific characteristics. In a CAT, the student starts with a question based on a single patient from his own case load and tries to answer this by searching scientific articles as is done in a systematic review. Findings are used for treatment of one specific patient. This knowledge is without doubt necessary throughout the working life of any medical doctor or paramedical professional. Assessment of study quality of observational studies is important not only in systematic reviews but also in CATs. In particular for CATs, study quality lists should be short and easy to understand.

Several criteria lists for observational studies are available (8). However, because they could not be used for the above mentioned systematic reviews, the need for a new methodological quality list becomes imperative. The Newcastle-Ottawa Scale (NOS) (5) is most widely used (e.g. (17; 18)). However, the NOS has various disadvantages. First, the NOS has a list for cohort and for case-control studies but not for cross-sectional studies. Second, lay raters get lost in the staccato terminology and lay-out. Third, the NOS is based on old cohort studies following a group of exposed and a group of non-exposed, while recent cohort studies usually assess multiple exposures in one population (13). So, the NOS is outdated when scoring recent cohort studies. In addition, the NOS has never been published in a peer reviewed journal. For this reason, date of origin is unknown. The first systematic review in Pubmed using the NOS is published in 2003 (19). Finally, an article criticizing the NOS pointed at some limitations, mainly in the case-control criteria, that could easily be solved (20). For example, the NOS rates independent case-ascertainment by two reviewers and blind assessment of exposure, while with respect to these two items validity in general is more important. In the results section of the present paper these issues are addressed.

The Strengthening the Reporting of Observational Studies in Epidemiology (Strobe) is a 22-item checklist designed for authors of observational studies to improve the quality and generalizability of observational research (6). It is not designed as a methodological quality list. Because there is no consensus what criteria list to use, the Strobe is used as a methodological criteria list (e.g. see (21; 22; 23)). The main disadvantage of the Strobe is that all items prescribe where in the text information should be provided. This increases readability of the articles, but not all items are applicable for methodological quality. In addition, even items that are related to methodological quality aim at reporting rather than methodological quality (e.g. “Clearly define all exposures” while “Is the assessment of the main independent variable valid?” would score quality). Therefore, the Strobe is not deemed efficient to assess the quality of observational studies.

Recently, a new criteria list for all NRS regardless of study design was developed, the ROBINS-I (9). After three years of expert meetings and feedback the final instrument was ready. ROBINS-I includes several domains and each domain starts with a signaling question. Although the ROBINS-I is designed for NRS, the rater starts with a “target RCT” studying the same research question. Bias is the expected difference between the hypothetically performed target RCT and the NRS of interest. Therefore, the terminology used, the description and elaboration of the bias, is as if judging RCTs. For example, the term “intervention” actually means “exposure” (9). Other disadvantages are: (a) Although developed for all study designs, the ROBINS-I is especially useful for studies with cohort-like designs. It is likely that modifications are desirable for other study types (9). (b) As the ROBINS-I is very detailed, using it is very complicated and time-consuming. (c) Well-known fallacies and flaws of several study designs (13; 24) are not included. For example, exposure to the independent variable, exclusion of subjects where outcome is present at baseline and length of follow-up. In the discussion, more details are provided.

The above-mentioned disadvantages of the NOS, Strobe and ROBINS-I prevented us from using one of these quality lists for our planned meta-analyses. Earlier, 80 observational study quality lists were found of which none was identified as the single comprehensive quality assessment list (25; 26). Because not all raters of observational studies develop their own quality list as suggested in this earlier overview (25), a short but universal quality list for future meta-analyses, systematic reviews and CATs using observational studies is needed.

Consequently, aim of the present paper is to compose, a comprehensive and widely usable quality criteria list for observational studies; the Observational Study Quality Evaluation (OSQE). Two above-mentioned criteria lists for observational studies (STROBE and NOS) (5; 6) and a criteria list for RCTs (1) serve as a basis for the OSQE. In addition, other criteria lists are checked to find additional items. The OSQE intends to compare methodological quality of studies using the same study design, as opposed to the ROBINS-I. Quality scores can be used to perform sensitivity analyses excluding poor quality studies or can be included as a modifier in meta-regression analysis. The OSQE includes separate quality lists for cohort studies (OSQE cohort), case-control studies (OSQE case-control) and cross-sectional studies (OSQE cross-sectional). The OSQE assesses methodological quality only. For guidelines how to perform and report a systematic review, the Preferred Reporting Items for Systematic Reviews and Meta-Analyses (PRISMA) is recommended (27).

1. **Methods**

All items of the NOS and the Strobe (5; 6) (observational studies) and non-RCT items of a criteria list for RCTs (1) were combined in a new list. The three lists are described below.

The **NOS** (5) consists of two checklists one for cohort studies and one for case-control studies. It includes items on case definition (case-control studies), exposure assessment, and representativeness. The full criteria list can be obtained via a website (5).

The **Strobe** (6) consists of 18 items for cohort, case-control and cross-sectional studies and three items that are specific for one of those three. Introduction, method, results and discussion sections of an article each have a set of items.

Although an RCT is a different type of research and not all items of an RCT-checklist are applicable, checking usefulness of the items of an RCT-criteria list can help when designing a new criteria list. For development of the OSQE, the **10-item criteria list for RCTs** used in CAT education in Maastricht was selected (1). This list was based on other criteria lists (28; 29). All items that are applicable to observational studies were selected (e.g. representativeness of the study population, impact of confounders and loss to follow-up).

When integrating the three above-mentioned methodological criteria lists, various stages were completed. First, items from the three lists were combined and language was improved. Reporting criteria and items only applicable to RCTs were removed. Second, the information sheet was added (see 3.1). Third, an additional file with clear explanation per item was written. Furthermore, all other available methodological quality criteria lists for observational studies were checked for additional items (see discussion of the present paper). Subsequently, the OSQE including the additional file was piloted in 7 raters; they gave feedback to the epidemiologist. Where needed the OSQE and explanation file were revised. Finally, two sets of raters rated articles for systematic reviews independently in order to obtain reliability (Pearson correlation).

1. **Results**

Figure 1 and 2 provide the OSQE cohort and the OSQE case-control, respectively. The OSQE cross-sectional includes a selection of the OSQE cohort items (see below). An excel file including the OSQE cohort, OSQE case-control and the OSQE cross-sectional is available in the additional material. All criteria of all three included criteria lists (**10-item criteria list for RCTs, NOS and Strobe;** (1; 5; 6)) were included with a few exceptions. First, criteria prescribing the section of the article where something should be described (reporting criteria; e.g. in the Strobe) were omitted, with the exception of four items at the end of the OSQE. Those four items were not included in the scoring, but could help obtain insight in the quality of reporting. Second, criteria specific for an RCT such as randomization and blinding from the RCT-criteria (1) were excluded from the pool of items. Finally, one item of the Strobe-items (provide study design early in methods) was extended to reflect a concept that would otherwise be missing (item 14 “Did the reporting of the results follow a protocol? In other words, were only *a priory* intended analyses reported?”). This item is included in other criteria lists, as presented in the discussion (9; 30).

<<please insert figure 1 and 2>>

The OSQE includes multiple-choice items. For each item the rater has to add qualitative comments. All items also include the answer “unknown”. Raters check this answer when the answer is not provided in the article or any other article presenting the same study (e.g. an earlier methods-article). The OSQE items are rather short so that the rater can add notes. Extra explanation with each item is included in a separate file (additional file 1: extra explanation). Analogue to the NOS (5), each question receives a star when the most optimal answer is given. It is possible that authors of an original study made a crucial error. In that case, the study quality is poor despite the number of stars obtained at the other items. Therefore, the OSQE also includes a veto column. Checking the veto column automatically places the article in the low-validity group, despite any stars on other items.

***3.1 Information sheet***

Because OSQE items are concise and universal, specification of the items is needed depending on the research question. Thus, before starting the scoring process, the rater needs to define how to score all included studies. For this reason, an information sheet is added to the OSQE (table 1 and 2, first excel sheet in the OSQE excel file, additional information). Various items have predefined questions. Raters can add information explaining any other item if needed. When performing a systematic review or meta-analysis, it is recommended that two or more raters reach consensus, also in agreement with PRISMA (27). In addition, the information sheet needs to be filled in transparently. When scoring only one or two observational studies (e.g. for a CAT), this process could be more implicit.

<<please insert table 1 and 2>>

***3.2 Cohort studies***

The OSQE cohort is presented in figure 1 and in the additional material. The OSQE cohort includes 14 obligatory items. In addition, two items are optional. First, when effect modification is likely in the included original studies, an extra item should be checked (item 15). Second, when raters are not going to perform a meta-analysis, sample size should be rated (item 16). In a meta-analysis, outcomes of the studies are weighted taking into account the sample size making this item redundant. Thus, original studies can obtain up to 14, 15, or 16 stars.

***3.3 Case-control studies***

The OSQE case-control (figure 2 and additional material) also includes 14 obligatory items and 2 optional items. Optional items are the same as in the OSQE cohort. Items 2, 6, 7, 9 and 10 are different from the OSQE cohort version. These items enquire whether cases and controls stem from the same source population, ascertainment of cases and controls, disease free controls, response and differential response between cases and controls.

***3.4 Adapt case-control list to meet earlier critique***

Earlier, the case-control version of the NOS was criticized (20). Below the critiques are addressed, consecutively. First, the NOS item on ***case-ascertainment*** in case-control studies is interpreted differently in the OSQE (question 6). In the NOS, case-ascertainment by two independent researchers was crucial. Instead, the OSQE asks for validity of case-ascertainment in general to be specified by the rater. This way the critique on the NOS is incorporated (20). Second, in the NOS, case-control studies with ***hospital controls*** do not obtain a star. This is the same in the OSQE (item 2), while the critique does imply that studies using hospital controls do obtain a star (20). Despite hospital controls do stem from the same source population, the use of this type of controls can introduce bias. For example, when patients with a broken leg are selected as hospital controls in a lung-cancer study, this may lead to the false conclusion that performing sports protects against lung cancer. In general terminology, the reason why controls are admitted to the hospital seems to protect for being a case (13; 24). In addition, when hospital controls are suffering from a disease with the same risk factor as the disease under study, the risk factor is biased towards no association (13; 24). Finally, the OSQE judges validity of the ***assessment of exposure*** more important than blinding the assessors for case-status (item 3). This is in agreement with the critique (20).

***3.5 Cross-sectional studies***

A subset of the OSQE cohort can be used to score cross-sectional studies, i.e. items 1, 3, 4, 5, 12, 13, 14 (optional 11, 15 and 16, see additional material). Items with respect to follow-up and exclusion of subjects at baseline are not applicable and thus validity of this study design is intrinsically lower. However, the other items can be scored enabling comparison of methodological quality within a group of cross-sectional studies. For example, items focusing on representativeness and confounding remain important.

***3.6 Reliability***

In running meta-analyses reliability of the OSQE scores was analyzed. Pearson correlation coefficient of OSQE cohort and OSQE case-control was r=0.71 (n=45) and r=0.80 (n=8), respectively (Anonymous, submitted). In another meta-analysis, Pearson correlation coefficient of OSQE case-control was r=0.81 (n=21) and r=0.51 (n=11), respectively (two second raters) and Pearson correlation coefficient of OSQE cross-sectional was r=0.65 (n=7), respectively (Anonymous, in prep). Neither of the raters used a cut-off point.

1. **Discussion**

The OSQE provides a comprehensive and widely applicable list for the assessment of study quality in observational studies. The OSQE is based on existing quality assessment lists. All items are included, but items that are criticized in the literature have been adapted. Each item has a comments field and those qualitative comments are most important. However, stars as well as the sum of stars are included to have a rough tool to discriminate study quality.

***4.1 Points of attention for raters***

Both users of the OSQE and raters of other methodological quality lists should comply with various general guidelines. First, when performing a systematic review or meta-analysis, raters should file the marked articles for reasons of transparency (25). Second, specification of criteria how each item should be scored is crucial for every research question. For this, the information sheet is added to the OSQE excel file (table 1). Third, initially none of the eligible studies should be excluded from a meta-analysis because of poor methodological quality. A sensitivity analysis can be performed excluding the poor-quality studies (sub group analysis). Alternatively, a dichotomous or categorical study quality variable can be added as a modifier to a meta-regression analysis. When multiple observational studies all have the same methodological problem, this flaw can be analyzed separately (presence or absence of the flaw as a modifier), because it has been shown that this can impact the results (31). Finally, it is advised not to use weights based on methodological quality (25; 31). Reason is that when summing the stars all criteria are considered equally important, while this is not the case. Weighting cannot solve this problem because all weights are arbitrary. Therefore, a general cut-off point for the number of stars to discriminate between good and poor study quality is not provided. For above mentioned sensitivity analysis or inclusion of a moderator, the rater can determine the optimal cut-off point. By including all vetoed studies in the poor-quality category, categorization of the studies better reflects real study quality.

***4.2 Other quality lists for observational studies***

There is more literature on study quality/risk of bias than the lists used in the present paper. Various literature was scrutinized to improve the OSQE. First, the critical appraisal tools provided by the Joanna Briggs Institute of the University of Adelaide included lists for three types of observational studies: cohort, case-control and cross-sectional studies (32). The Joanna Briggs Institute cohort list included one item that was not included in the OSQE, i.e. similarity of exposure assessment in exposed and unexposed subjects. The assumption was that only a minority of the recent cohort studies include a cohort with exposed and another cohort with unexposed subjects. For this reason, this item was not added to the OSQE. Additionally, the Joanna Briggs case-control list included four items that were different from the OSQE, an item on matching, an item on the comparability of the groups, an item on control for confounders and an item on the appropriateness of the analyses. Because of overlap between those four, the OSQE combined all in one item (item 13). An item whether “the same criteria were used for identification of cases and controls” was not included in the OSQE. This can be scored as part of item 6 (adequate case definition).

Second, “Conducting Systematic Reviews and Meta-Analyses of Observational Studies of Etiology” (COSMOS-E) provides a set of seven principles to comply with when assessing quality of observational studies (25). The OSQE complies with the COSMOS-E principles. For example, principle 4 argues that risk of bias should be assessed per outcome and principle 5 prescribes that the article copies used for the scoring should be filed for transparency. To comply with principle 7, the COSMOS-E tip to think of the perfect study is added to the additional file. Both the COSMOS-E (principle 2) and the OSQE stress the importance of qualitative comments. Despite that the OSQE does include a sum of stars trying to discriminate between good and poor quality (against principle 6 of the COSMOS-E: “summary score should be avoided” (25)). Otherwise, analyzing whether the results are different depending on the quality of the study is not possible. A veto column categorizing a single study as poor quality whatever stars it received on any of the other items partly removes the drawbacks highlighted in the COSMOS-E. Principle 1 suggests that a universal criteria list as the OSQE is impossible. Instead, areas to be scored should be selected for each study domain separately (25). It is unlikely that researchers, medical doctors and students generate a new quality list for each CAT, systematic review, meta-analysis or assignment, for reasons of time constraints and limited methodological expertise. To comply with principle 1 of the COSMOS-E, the OSQE includes an information sheet with specific questions per item that the rater should fill in before scoring articles for that specific research question (table 1) (25).

Third, the quality index developed by Downs and Black (30) (hereafter D&B) aimed to score both RCT and NRS using the same instrument. D&B pleas for the use of sub-scales; the authors argue that authors of NRS should discuss consequences of weaknesses rather than only generating a sum score. For the same reason, the OSQE encourages qualitative assessment. Both D&B and OSQE include external validity of the study, as opposed to the ROBINS-I. While D&B includes separate items for internal and external validity, the OSQE asks the rater to judge the balance between internal and external validity. This is in agreement with the 10-item criteria list for RCTs (1) and with the fact that an increase in internal validity always goes at the expense of external validity and vice versa (13). As the Strobe, D&B includes reporting items in their quality index sum score. In 1998, techniques for meta-analysis were still in their infancy and thus the authors still included power and even suggested to give less weight to null-findings in small studies. Nowadays this would be considered incorrect. Because of publication bias, null-findings are often underrepresented (33). When performing a meta-analysis, sample size / power does not need to be scored because the meta-analysis generates a result weighted by the number of subjects. The OSQE does include an optional item on sample size / power. As soon as the rater does not perform a meta-analysis, this item should be scored. Except for reporting items, power and RCT items, all D&B items are also included in the OSQE.

Furthermore, the Critical Appraisal Skills Programme (CASP) provided lists for cohort and case-control studies (8; 34). The CASP case-control and cohort were designed for medical students. It was better suited for the use in education, than for the use in systematic reviews. The CASP missed a lot of items that were included in other methodological criteria lists (internal validity-external validity, specific flaws for cohort case-control designs, missing data). The CASP did include various items that were not in the OSQE. However, these do not belong in a methodological quality list (e.g. “What are the results of this study”, “do you believe the results” and “what are implications of this study for practice”). In addition, the screening questions were too broad including multiple topics combined. National Heart Lung and Blood Institute (NIH) and Scottish Intercollegiate Guidelines Network (SIGN) were rather similar to the OSQE lists, with some minor differences (35; 36).

Finally, the ROBINS-I was developed recently. Table 3 presents differences between OSQE and ROBINS-I in more detail. The ROBINS-I intends to harmonize scoring between study designs (RCT, cohort, case-control, cross-sectional, case series and case reports), while the OSQE aims to assess study quality within studies sharing the same study design (e.g. cohort studies only). When a meta-analysis includes both RCTs and NRS’, ROBINS-I could be a better choice. However, it is also possible to use a different criteria list for each study design and add the variable “study design” as a modifier to the meta-analysis. While the ROBINS-I is extensive and difficult to score for some raters, the OSQE aims to be both comprehensive and comprehensible for raters with various levels of expertise. The OSQE includes some items that the ROBINS-I does not include (table 3). Another difference is that the OSQE emphasizes the importance of qualitative information, while the ROBINS-I only asks for quantitative scoring (9; 14).

<<please insert table 3>>

Other recent methodological quality criteria lists were generated after extended periods of expert meetings or by performing factor analysis (3; 9). Despite the overlapping items, available methodological quality lists for observational studies were not suitable for the above-mentioned systematic reviews (Anonymous, submitted, Anonymous, in prep). For this reason, the OSQE combined existing methodological quality lists, rather than going through the process to generate a new criteria list from scratch. A limitation is that the OSQE is put together by a single epidemiologist. However, exclusion of items was minimal and is transparently explained in the present paper. When checking all other existing criteria lists, no other items were found. Extra attention was paid to readability and understandability. For this the OSQE was piloted. This way, an instrument that is suitable for consensus, reliable and broadly applicable, while also available to be used in our meta-analyses, was created.

**Acknowledgements**

We thank Leonie Banning for commenting on both the OSQE (cohort, case-control and cross-sectional) and a draft of the article.

**Contribution to the field**

Although, plenty of instruments are available to assess methodological quality and risk of bias, for observational studies we missed the optimal instrument. NOS, Strobe and ROBINS-I and the most recommended instruments. However, NOS is not easy to score and outdated. Strobe is designed as a checklist for researchers performing an observational study. The recently developed ROBINS-I has several disadvantages: its extensiveness hampers use in large meta-analyses, not suitable for cross-sectional studies, the search for analogy with RCTs using RCT terminology results in an unnecessary complicated checklist while omitting well-known flaws in observational studies. Therefore, a comprehensive and understandable methodological quality / risk of bias list is highly needed.

We developed the Observational Study Quality Evaluation (OSQE). Systematic reviews and meta-analyses build on observation studies are important additions to science. Observational studies have their merits and are published in large quantities. These data cannot not be neglected.

Both ROBINS-I and OSQE have their own qualities. Raters can choose either of them depending on the studies to be scored and their expertise.

**List of abbreviations**

CAT Critical Appraisal of a Topic

COSMOS-E Conducting Systematic Reviews and Meta-Analyses of Observational Studies of Etiology

D&B Downs and Black

OSQE Observational Study Quality Evaluation

NOS Newcastle-Ottawa Scale

RCT Randomized controlled trail

ROBINS-I Risk Of Bias In Non-randomized Studies – of Interventions assessment tool

Strobe Strengthening the Reporting of Observational Studies in Epidemiology

**Ethics approval and consent**

Not applicable. The present paper did not use human participants or animal data. Thus, neither ethical approval nor informed consent were needed.

**Availability of data**

Reliability data are included in the submission as supplementary material.

**Competing interests**

The authors declare that they have no competing interests

**Funding**

None

**Authors' contributions**

MD generated the OSQE and wrote the manuscript. IW, CvH and EV used the OSQE to score observational studies, commented and improved the OSQE and revised the manuscript. MB commented and improved the OSQE and revised the manuscript. All authors read and approved the final version of the manuscript.

**Supplementary material**

File name: Additfile1.docx

Format: Word (.docx)

Title: Explanation of each OSQE item

Description: Explanation of each OSQE item

File name: Additfile2.xlsx

Format: Excel (.xlsx)

Title: Excel file with the OSQE (cohort, case-control and cross-sectional), including colors and formulae

Description: The OSQE

File name: Additfile3.xlsx

Format: Excel (.xlsx)

Title: reliability data

Description: In duplo ratings of OSQE cohort, OSQE case-control, OSQE cross-sectional (Anonymous, submitted, Anonymous, in prep).

**References**

1. C. De Brouwer, M. Mommers, C. Van Gool, I. Kant, and I. Ferreira, Training Critical Appraisal of a Topic; an indispensable manual in the area of Evidence Based Medicine, Maastricht, Mediview (2012).

2. J.P. Higgins, D.G. Altman, P.C. Gotzsche, P. Juni, D. Moher, A.D. Oxman, J. Savovic, K.F. Schulz, L. Weeks, J.A. Sterne, G. Cochrane Bias Methods, and G. Cochrane Statistical Methods, The Cochrane Collaboration's tool for assessing risk of bias in randomised trials. BMJ (2011) 343: d5928.

3. B.J. Shea, J.M. Grimshaw, G.A. Wells, M. Boers, N. Andersson, C. Hamel, A.C. Porter, P. Tugwell, D. Moher, and L.M. Bouter, Development of AMSTAR: a measurement tool to assess the methodological quality of systematic reviews. BMC Med Res Methodol (2007) 7: 10.

4. B.J. Shea, C. Hamel, G.A. Wells, L.M. Bouter, E. Kristjansson, J. Grimshaw, D.A. Henry, and M. Boers, AMSTAR is a reliable and valid measurement tool to assess the methodological quality of systematic reviews. J Clin Epidemiol (2009) 62: 1013-20.

5. G. Wells, B. Shea, D. O'Connell, J. Peterson, V. Welch, M. Losos, and P. Tugwell, The Newcastle-Ottawa Scale (NOS) for assessing the quality of nonrandomised studies in meta-analyses: The Ottawa Hospital Research Institute, (Ed.) (unknown) <http://www.ohri.ca/programs/clinical_epidemiology/oxford.asp>, [Accessed August 2019].

6. J.P. Vandenbroucke, E. von Elm, D.G. Altman, P.C. Gotzsche, C.D. Mulrow, S.J. Pocock, C. Poole, J.J. Schlesselman, M. Egger, and S. initiative, Strengthening the Reporting of Observational Studies in Epidemiology (STROBE): explanation and elaboration. Ann Intern Med (2007) 147: W163-94.

7. M. Salzmann-Erikson, and J. Dahlen, Nurses' Establishment of Health Promoting Relationships: A Descriptive Synthesis of Anorexia Nervosa Research. J Child Fam Stud (2017) 26: 1-13.

8. L.L. Ma, Y.Y. Wang, Z.H. Yang, D. Huang, H. Weng, and X.T. Zeng, Methodological quality (risk of bias) assessment tools for primary and secondary medical studies: what are they and which is better? Mil Med Res (2020) 7: 7.

9. J.A. Sterne, M.A. Hernan, B.C. Reeves, J. Savovic, N.D. Berkman, M. Viswanathan, D. Henry, D.G. Altman, M.T. Ansari, I. Boutron, J.R. Carpenter, A.W. Chan, R. Churchill, J.J. Deeks, A. Hrobjartsson, J. Kirkham, P. Juni, Y.K. Loke, T.D. Pigott, C.R. Ramsay, D. Regidor, H.R. Rothstein, L. Sandhu, P.L. Santaguida, H.J. Schunemann, B. Shea, I. Shrier, P. Tugwell, L. Turner, J.C. Valentine, H. Waddington, E. Waters, G.A. Wells, P.F. Whiting, and J.P. Higgins, ROBINS-I: a tool for assessing risk of bias in non-randomised studies of interventions. BMJ (2016) 355: i4919.

10. D.C. Grootendorst, K.J. Jager, C. Zoccali, and F.W. Dekker, Observational studies are complementary to randomized controlled trials. Nephron Clin Pract (2010) 114: c173-7.

11. N. Black, Why we need observational studies to evaluate the effectiveness of health care. BMJ (1996) 312: 1215-8.

12. J.P. Vandenbroucke, When are observational studies as credible as randomised trials? Lancet (2004) 363: 1728-31.

13. K.J. Rothman, and T. Lash, Modern epidemiology, Lippincott Williams And Wilkins (2018).

14. H.J. Schunemann, C. Cuello, E.A. Akl, R.A. Mustafa, J.J. Meerpohl, K. Thayer, R.L. Morgan, G. Gartlehner, R. Kunz, S.V. Katikireddi, J. Sterne, J.P. Higgins, G. Guyatt, and G.W. Group, GRADE guidelines: 18. How ROBINS-I and other tools to assess risk of bias in nonrandomized studies should be used to rate the certainty of a body of evidence. J Clin Epidemiol (2019) 111: 105-114.

15. J. Albrecht, V.P. Werth, and M. Bigby, The role of case reports in evidence-based practice, with suggestions for improving their reporting. J Am Acad Dermatol (2009) 60: 412-8.

16. J.P. Vandenbroucke, [The importance of case reports as compared to evidence-based medicine and molecular explanation]. Ned Tijdschr Geneeskd (2002) 146: 1699-703.

17. L.C.P. Banning, I. Ramakers, K. Deckers, F.R.J. Verhey, and P. Aalten, Apolipoprotein E and affective symptoms in mild cognitive impairment and Alzheimer's disease dementia: A systematic review and meta-analysis. Neurosci Biobehav Rev (2019) 96: 302-315.

18. J.T. Henderson, E.M. Webber, and S.I. Bean, Screening for Hepatitis B Virus Infection in Pregnant Women: An Updated Systematic Review for the U.S. Preventive Services Task Force, Screening for Hepatitis B Virus Infection in Pregnant Women: An Updated Systematic Review for the U.S. Preventive Services Task Force, Rockville (MD), (2019).

19. J.J. Deeks, J. Dinnes, R. D'Amico, A.J. Sowden, C. Sakarovitch, F. Song, M. Petticrew, D.G. Altman, G. International Stroke Trial Collaborative, and G. European Carotid Surgery Trial Collaborative, Evaluating non-randomised intervention studies. Health Technol Assess (2003) 7: iii-x, 1-173.

20. A. Stang, Critical evaluation of the Newcastle-Ottawa scale for the assessment of the quality of nonrandomized studies in meta-analyses. Eur J Epidemiol (2010) 25: 603-5.

21. K.W. Lee, S.M. Ching, V. Ramachandran, A. Yee, F.K. Hoo, Y.C. Chia, W.A. Wan Sulaiman, S. Suppiah, M.H. Mohamed, and S.K. Veettil, Prevalence and risk factors of gestational diabetes mellitus in Asia: a systematic review and meta-analysis. BMC Pregnancy Childbirth (2018) 18: 494.

22. T.R. Stanton, H.B. Leake, K.J. Chalmers, and G.L. Moseley, Evidence of Impaired Proprioception in Chronic, Idiopathic Neck Pain: Systematic Review and Meta-Analysis. Phys Ther (2016) 96: 876-87.

23. A. Umer, G.A. Kelley, L.E. Cottrell, P. Giacobbi, Jr., K.E. Innes, and C.L. Lilly, Childhood obesity and adult cardiovascular disease risk factors: a systematic review with meta-analysis. BMC Public Health (2017) 17: 683.

24. K.J. Rothman, and S. Greenland, Modern Epidemiology, Philadelphia, Lippincott-Raven (1998) p. 738.

25. O.M. Dekkers, J.P. Vandenbroucke, M. Cevallos, A.G. Renehan, D.G. Altman, and M. Egger, COSMOS-E: Guidance on conducting systematic reviews and meta-analyses of observational studies of etiology. PLoS Med (2019) 16: e1002742.

26. S. Sanderson, I.D. Tatt, and J.P. Higgins, Tools for assessing quality and susceptibility to bias in observational studies in epidemiology: a systematic review and annotated bibliography. Int J Epidemiol (2007) 36: 666-76.

27. D. Moher, A. Liberati, J. Tetzlaff, D.G. Altman, and P. Group, Preferred reporting items for systematic reviews and meta-analyses: the PRISMA statement. J Clin Epidemiol (2009) 62: 1006-12.

28. D. Badendoch, and C. Heneghan, Evidence based medicine toolkit, London, BMJ books (2002).

29. M. Offringa, W. Assendelft, and R. Scholten, Inleiding in de evidence based medicine; klinisch handelen gebaseerd op bewijsmateriaal. [Introduction to evidence based medicine; basing clinical actions on the evidence., Houten, Bohn Stafleu van Loghum (2008).

30. S.H. Downs, and N. Black, The feasibility of creating a checklist for the assessment of the methodological quality both of randomised and non-randomised studies of health care interventions. J Epidemiol Community Health (1998) 52: 377-84.

31. D.F. Stroup, J.A. Berlin, S.C. Morton, I. Olkin, G.D. Williamson, D. Rennie, D. Moher, B.J. Becker, T.A. Sipe, and S.B. Thacker, Meta-analysis of observational studies in epidemiology: a proposal for reporting. Meta-analysis Of Observational Studies in Epidemiology (MOOSE) group. JAMA (2000) 283: 2008-12.

32. Joanna Briggs Institute University of Adelaide, Critical appraisal tools (2017) <https://joannabriggs.org/ebp/critical_appraisal_tools>, [Accessed October 2019].

33. H. Sharma, and S. Verma, Is positive publication bias really a bias, or an intentionally created discrimination toward negative results? Saudi J Anaesth (2019) 13: 352-355.

34. Critical Appraisal Skill Programme, CASP Cohort and CASP case-control checklist (2018) <https://casp-uk.net/casp-tools-checklists/>, [Accessed February 2021].

35. National Heart Lung and Blood Institute, Study Quality Assessment Tools (unknown) <https://www.nhlbi.nih.gov/health-topics/study-quality-assessment-tools>, [Accessed February 2021].

36. Scottish Intercollegiate Guidelines Network, Methodology Checklist 3: Cohort studies (unknown) <https://www.sign.ac.uk/what-we-do/methodology/checklists/>, [Accessed February 2021].

37. L.D. Claxton, A review of conflict of interest, competing interest, and bias for toxicologists. Toxicol Ind Health (2007) 23: 557-71.

38. M. Wilson, The New England Journal of Medicine: commercial conflict of interest and revisiting the Vioxx scandal. Indian J Med Ethics (2016) 1: 167-71.

**Figure 1. The OSQE (cohort)**

NOTE. This is a copy of an excel file (additional file 2). In the excel file cells in yellow are filled in automatically, based on information in other cells. The check-column to the right is checked when the item is filled in. This way the researcher is pointed at missing items.

The following references were used to compose the OSQE: (1; 5; 6).

**Figure 2. The OSQE (case-control)**

NOTE. This is a copy of an excel file (additional file 2). In the excel file cells in yellow are filled in automatically, based on information in other cells. The check-column to the right is checked when the item is filled in. This way the researcher is pointed at missing items.

The following references were used to compose the OSQE: (1; 5; 6).

Table 1. Information sheet; several questions to be answered before scoring the OSQE cohort

| ***Information sheet*** | |
| --- | --- |
|  | ***Raters performing a systematic review or meta-analysis: please answer the questions below before scoring the OSQE*** |
|  | **File the marked articles !** |
|  | ***Answer the questions for your review, not for each paper separately. E.g. your main dependent variable can be a secondary outcome in the paper, but still you have to score validity of that outcome. CAVE: numbers correspond with the numbers in the OSQE.*** |
|  | ***For items 1, 3, 4, 5, 8, 10, 13 and 15 explanation is obligatory. Information needed for other items can be added by inserting extra rows.*** |
| 1 | This item of the OSQE searches for a good balance between internal and external validity. Please define which in- and exclusion criteria are OK and which are not. This depends on your choice for this balance in the articles in your meta-analysis. |
| 1 | A specific question: what response rate is acceptable? When response rate is low, representativeness is not OK. |
| 3 | What are the main independent variables? |
| 4 | How is optimal exposure defined? |
| 5 | What are the dependent variables of interest? (Also consider co-interventions) |
| 8 | What is the minimum follow-up duration that you think is adequate? (*Assuming average follow-up, when follow-up duration varies*) |
| 10 | Loss-to-follow-up lower than 10% does not introduce bias. For your systematic review: do you think this 10% is the correct cut-off. If not change. |
| 13 | Which confounders are relevant? |
| 15 | Are there any hypotheses of effect modification? If yes, what is the effect modifier? Please include question 14 in the scoring. |

Table 2. Information sheet; several questions to be answered before scoring the OSQE case-control

|  | ***For items 1, 3, 4, 6, 7, 8, 9, 13 and 15 explanation is obligatory. Information needed for other items can be added by inserting extra rows.*** |
| --- | --- |
| 1 | This item of the OSQE searches for a good balance between internal and external validity. Please define which in- and exclusion criteria are OK and which are not. This depends on your choice for this balance in the articles in your meta-analysis. |
| 1 | A specific question: what response rate is acceptable? When response rate is low, representativeness is not OK. |
| 3 | What are the main independent variables? |
|  | How likely is recall bias with this research question? |
| 4 | How is optimal exposure defined? |
| 6 | What criteria are defined to select cases? |
| 7 | Does the control group need to be disease free? (if not omit item 7) |
| 8 | What is the minimum duration between exposure and outcome that you think adequate? |
| 9 | Cut-offs for fair, good, excellent response could be 60 and 90%. The rater needs to define what response percentage is excellent in this area of research. |
| 13 | Which confounders are relevant? The rater has to keep in mind that even matching variables should be controlled for in the analysis. |
| 15 | Are there any hypotheses of effect modification? If yes, what is the effect modifier? Please include question 14 in the scoring. |

Table 3. Comparison between ROBINS-I and OSQE

***Section A: Overlap between ROBINS-I and OSQE^1^***

| ROBINS-I | Bias due to ... | OSQE Cohort | OSQE Case-Control |
| --- | --- | --- | --- |
| Pre intervention | .. confounding | 13 | 13 |
|  | .. selection of participants | 1 | 1 |
| At intervention | .. classification of interventions | 3 | 3 |
| Post-intervention | .. deviation from intended interventions | 4^2^ | 4^2^ |
|  | .. missing data | 10, 11 | 9, 11 |
|  | .. measurement of the outcomes | 5, 6 | 6 |
|  | .. selection of the reported results | 14 | 14 |

^1^ ROBINS-I and OSQE have in common that raters are asked to define several items, before starting to rate. For example, the dependent and independent variables of interest. ROBINS-I additionally asks for research question and PICO (patient, intervention, comparison, outcome).

^2^ Similar but not the same

***Section B: OSQE items that were not in ROBINS-I***

| **OSQE cohort** | | **Reason for inclusion in OSQE** |
| --- | --- | --- |
| 2 | Inclusion of cohorts from multiple source populations | In older cohort studies this did happen and this could introduce bias. |
| 4 | Presence of the independent variable | ROBINS-I enquires deviation from the intended intervention (even including balance between intervention groups). OSQE item 4 is broader (all reasons why exposure is lower) and does not enquire balance between groups (assuming that control groups not always have an alternative exposure). |
| 7 | Exclusion of subjects were outcome is present at baseline | Obviously, in an RCT those subjects are also excluded, but criteria lists for RCTs do not include this criterion. In a cohort study, the likeliness of this is much higher. |
| 8 | Follow-up sufficiently long | When follow-up is too short outcome may not have occurred. |
| 9 | Continuous assessment of outcome | As opposed to RCTs, longitudinal observational studies sometimes have no follow-up assessments, but instead use existing registrations and databases. |
| 12 | Conflict of interest | In criteria lists for both RCTs and observational studies, there is a debate whether or not to include conflict of interest as a criterion. The OSQE includes the item because it was in the NOS. It has been reported that conflict of interest is associated with biased (37; 38) |
| 14 | Effect modification | When there is a hypothesis for interaction, ignoring this would lead to erroneous results (13). |
| 15 | Sample size | Because the ROBINS-I is designed for meta-analyses only. Raters using the OSQE are instructed to omit this item when performing a meta-analysis. |
| **OSQE case-control** | |  |
| 2 | Cases and controls stem from different populations | Because of the difficulty to define the source populations of cases it is not obvious that cases and controls stem from the same population and this can introduce bias. In addition, the use of hospital controls can also introduce bias. |
| 4 | Presence of the independent variable | See OSQE Cohort |
| 5 | Assessment of independent variable the same in cases and controls | In case-control studies data collection is often different in cases and controls. |
| 7 | Do controls have a history of the disease | When taking a random sample of the healthy population or when matching with the healthy population, a percentage similar to the prevalence of the disease of interest will have the disease. |
| 8 | Follow-up sufficiently long | See OSQE Cohort |
| 10 | Non-response similar in cases and controls | It is very well likely that cases are much more motivated to take part in the study that healthy controls who have no interest in the disease of interest. |
| 12 | Conflict of interest | See OSQE Cohort |
| 14 | Effect modification | See OSQE Cohort |
| 15 | Sample size | See OSQE Cohort |

An extended elaboration on fallacies in case-control and cohort studies can be found in epidemiology books, such as: (13).
