# Supplementary material for: Aggression on the psychiatric ward: Prevalence and risk factors. A systematic review of the literature
Source: PLoS One. 2021 Oct 8;16(10):e0258346. doi: 10.1371/journal.pone.0258346 (PMC8500453; doi:10.1371/journal.pone.0258346)
Supplement: S1 Table — (DOCX) [file pone.0258346.s004.docx]

S1 Table

| **Author** | **N** | **Prevalence/incidence** |
| --- | --- | --- |
| Niu et al, 2019 (18) | 429 | 56% experienced physical violence in the past year |
| Yang et al, 2018 (19) | 245 | 95% of staff experienced some incident of workplace violence in the past year  82% experienced a physical attack in the past year |
| Al-Azzam et al, 2017 (35) | 262 | 67% experienced workplace violence in the past 12 months  Incidence was 146 per 1000 case-years  19.8% of participants were exposed to violence twice in the last year |
| Pekurinen et al, 2017 (21) | 923 | 65% experienced some sort of aggression in the past 12 months  38% experienced physical assault in the past 12 months |
| Bilici & Izci, 2016 (22) | 137 | 72% of nursing assistants and nurse/health officers were victim of assault in their career  52% of physicians were victim of assault in their career |
| Ridenour et al, 2015 (23) | 284 | 85% of nurses experienced at least 1 assault of patients in the 30 days of survey |
| Unsal et al, 2013 (24) | 441 | 70% experienced verbal violence from a patient and 48% experienced physical violence from a patient both in the last year |
| Khoshknab et al, 2012 (25) | 183 | Prevalence of workplace violence is 71% in the past year of which:  physical violence: 93%  verbal violence: 6% |
| Dvir et al, 2012 (26) | 204 | During residency training (5 years):  86% was threatened  71% was physically intimidated  25% was physically assaulted  1 year prevalence of physical assault was 11%. |
| Moylan, 2011 (27) | 110 | 80% had been assaulted ever in their career (ranging from 1-4 years) |
| Altinbas et al, 2011 (28) | 186 | During their professional life (0.1-31 years (SD=7.1 and median=3.7):  70.9% experienced verbal or physical assault, combination was most common: 48.4%  88% experienced verbal aggression  51% experienced physical violence: 16% threatening gestures, 22% strikes, kicks or pushing, 11% attack to others with mild-moderate physical injury, 2% attack to others with severe physical injury. |
| Chen et al 2008 (29) | 222 | One year prevalence of verbal abuse: 50.9%  One year prevalence of physical violence: 35.1% |
| Merecz et al, 2006 (30) | 78 | 100% of psychiatric nurses experience verbal aggression in the past year  79% of psychiatric nurses experience physical attacks and 64% experienced a physical attack several times a year, 15% monthly and 1.3% weekly |
| Privitera et al, 2005 (31) | 380 | Incidence:  - past 12 months: 8 assaults, 37 verbal  - 1-5 years ago: 19 assaults, 24 verbal  - 6-10 years ago: 17 assaults, 22 verbal  - > 10 years ago: 11 assaults, 11 verbal: rise between time frames is significant (p<.001, 0.13, 0.14) |
| Spokes et al. 2002 (32) | 108 | 99% experienced one or more incidents of violence at work in their career (mean=44 months, range=1 month to 28 years) |
| Sjostrom et al, 2001 (33) | 47 | Prevalence of violence towards staff= 3% (20 incidents) during 2 study periods of 6 weeks |
| Schwartz, 1999 (34) | 517 | During residency (5 years):  36% reported 636 physical assaults  73% reported 1884 threats |
