# Supplementary material for: Aggression on the psychiatric ward: Prevalence and risk factors. A systematic review of the literature
Source: PLoS One. 2021 Oct 8;16(10):e0258346. doi: 10.1371/journal.pone.0258346 (PMC8500453; doi:10.1371/journal.pone.0258346)
Supplement: S2 Table — (DOCX) [file pone.0258346.s005.docx]

S2 Table

| **Author (year)** | **n (number of patients)** | **Prevalence/incidence** |
| --- | --- | --- |
| Winje et al, 2018 (36) | 67 | 40% involved in incident of aggression |
| Menculini et al, 2018 (37) | 160 | 55% showed violent behaviour at admission |
| Mi et al, 2017 (38) | 1288 | 48% showed aggression |
| Ben-Zeev et al, 2017 (39) | 27 | 52% showed one event of violent behaviour |
| George et al, 2016 (40) | 272 | 10% showed verbal agitation  14% showed non-goal directed agitation  7% showed goal directed physical agitation |
| Gunenc & Dickens, 2015 (41) | 613 | 341 patients caused 1594 incident of verbal aggression  33% showed mild verbal aggression, 50% severe verbal aggression |
| Cho et al, 2014 (42) | 443 | 28% showed aggression |
| Calegaro et al, 2014 (43) | 110 | First 24 hours:  37% showed aggression  34% showed verbal aggression  9% showed physical aggression  10% showed aggression to object |
| Stewart, 2013 (44) | 522 | 270 patients caused 1398 incidents of verbal aggression  5.2 incidents per patient |
| Grenyer et al, 2013 (45) | 64 | Mean number of incidents per patient =3.75 (*SD*=2.54)  81% physical aggression  57% aggression against staff  21% caused injury |
| Dickens et al, 2013 (46) | 373 | 68% was other-directed aggression committed by 43% of the patients  10% patients were involved in 54% of all incidents |
| Van Dongen et al, 2012 (47) | 44 | Verbal aggression: 25%  Threats: 18.%  Physical aggression: 2%  Aggression towards objects: 9% |
| Ross et al, 2012 (48) | 522 | 52%: at least one incident of verbal aggression  28%: at least one incident of physical aggression to objects  21%: at least one incident of physical aggression to others |
| Cookson et al, 2012 (49) | 79 | 76% showed aggressive behaviour  65% was towards staff  63% verbal aggression and 22.8% physical aggression |
| Chukwujekwu & Stanley, 2011 (50) | 298 | 20% was aggressive  9.8 incidents per month |
| Kruger &Rosema 2010 (51) | 262 | 16% involved in one or more violent acts |
| Tenneij et al, 2009 (53) | 108 | 415 incidents by 56% of the patients |
| Nolan et al, 2009 (54) | 66 | 64% showed aggression  315 incidents: 77% verbal aggression, 31% physical aggression to people, 10% physical aggression to objects |
| Bowers et al, 2009 (55) | ? | Mean daily rate of verbal abuse: 2.41 (*SD*=1.40), aggression to objects: 0.48 (*SD*=0.21), physical violence to others: 0.33 (*SD*=0.14) |
| Biancosino et al, 2009 (56) | 1324 | 10% showed aggressive behaviour in group of hostile patients |
| Carr et al, 2008 (57) | 3877 | 21% of admissions involved in aggressive incident  0.55 incidents per month per occupied bed |
| Amore et al, 2008 (58) | 303 | Of patients showing verbal or physical aggression before admission: 25% showed physical aggression and 12% showed verbal aggression during the admission |
| Quanbeck et al, 2007 (59) | 298 | 11.2 assault per patient (*SD*=6.2)  56% by forensic patients |
| Ketelsen et al, 2007 (60) | 2210 | 8% involved in one incident or more  2.5 (*SD*=3.4) incidents per patient  52% caused one incident  8% caused 6 or more incidents |
| Goldberg et al, 2007 (61) | 76 | 43% of patients showed aggression during first two weeks of admission |
| Foster & Nijman, 2007 (62) | 149 | 0.8 incidents per day  33% of patient accounted for 61% of the incidents |
| Abderhalden et al, 2007 (63) | 2017 | 1.8 incident per 100 treatment days  13% caused one or more incidents  3% caused 50% of all incidents  In first 3 days 26% of incidents happened |
| El-Badri, 2006 (64) | 535 | 32% of incidents happened in the first day of admission  67% happened in first week  74% was involved in only one incident |
| Grassi et al, 2006 (65) | 2259 | 39% of patients were aggression  2.5 incident per patient |
| Erkiran et al, 2006 (66) | 237 | 52% of patients with dual disorder and 7% of patients with schizophrenia were involved in recent violent event |
| Nolan et al, 2005 (67) | 157 | 56% involved in one or more incidents  0-27 incidents per patients (total 408 incidents) |
| Ferguson et al, 2005 (68) | 212 | 51% involved in aggressive behaviour |
| Vevera et al, 2005 (86) | 404 | Prevalence of violence: 1949=35%, 1969: 45%, 1989: 33%, 2000: 44%  Increasing trend over the years when adjusting for length of observation: OR=1.21 (95% CI=0.99-1.47) |
| Chang, 2004 (69) | 111 | 41% was responsible for 224 incidents of aggression  2 (*SD*=5.1) incidents per patient (range 0-37) |
| Troisi et al, 2003 (70) | 80 | 25% involved in act of aggression in first week  61% of incidents happened in first 3 days of admission |
| Parkes, 2003 (71) | 1473 incidents | 21 incidents per month |
| Mellesdal, 2003 (72) | 934 | 1% accounted for 49% of all aggressive incidents  In 8% of admissions aggression took place  20% of incidents in first 24 hours  54% of incidents in first week |
| Fagan-Pryor et al, 2003 (73) | 92 | 45% witnessed patient-patient aggression and 40% witnessed patient-staff aggression |
| Bowers et al, 2003 (74) | 282 | 52% showed verbal aggression |
| Abeyasinghe, 2003 (75) | 92 | 1.3 incident per patient  2.5 incidents per week |
| Nijman, 2002 (6) | 149 | N=774 incidents (74% outwardly directed)  19% of patients caused 52% of incidents |
| Ehman et al, 2001 (79) | 78 | 7.38 assaults per week, 0.057 incidents per patient day  26% of patients accounted for 79% of aggression to objects, 85% of selfinjurious acts, 61% of verbal aggression  64% of patients were assaultive |
| Soliman & Reza, 2001 (77) | 257 | 19% of patients involved in incident  0.22 incidents per admission per year  14% of patients caused 48% of incidents |
| Grassi et al, 2001 (78) | 1534 | 8% involved in aggressive incident  Prevalence is 8%  1% of patients caused 42% of incidents  54% in first week of admission  28% in first 2 days of admission |
| Barlow et al, 2000 (82) | 1269 | 14% of patients were aggressive  40% of patients were responsible for 71% of aggressive acts.  Most violence happened in the first 2 days  On average 5.05 incidents per week happened |
| Steinert et al, 2000 (80) | 138 | 38% of patients caused 142 incidents (2.7 incident per patient) |
| Eaton et a, 2000 (81) | 52 admissions | 17 patients were violent in first month of admission  2 patients caused 34% of incidents |
| Steinert et al, 1999 (87) | 471 | 70% showed some form of aggressive behaviour  65% was aggressive against others |
| Krakowski et al, 1999 (83) | 96 | 46% was persistently violent (repeatedly aggressive with a decrease of less than 50% after first 18 days of admission) |
| Hardie, 1999 (84) | 196 | 20% of patients caused 73% of all incidents (n=2053) |
| Arango et al, 1999 (85) | 63 | 25% was physically violent  0.4 incident per patient (n=26 incidents) |
| SD: Standard Deviation |  |  |
|  | | |
